# Making Sense of Residues on Flaked Stone Artefacts: Learning from Blind Tests

**DOI:** 10.1371/journal.pone.0150437

**Published:** 2016-03-01

**Authors:** Veerle Rots, Elspeth Hayes, Dries Cnuts, Christian Lepers, Richard Fullagar

**Affiliations:** 1 TraceoLab / Préhistoire, University of Liège, Liège, Belgium; 2 Centre for Archaeological Science, University of Wollongong, Wollongong, Australia; Universidade do Algarve, PORTUGAL

## Abstract

Residue analysis has become a frequently applied method for identifying prehistoric stone tool use. Residues adhering to the stone tool with varying frequencies are interpreted as being the result of an intentional contact with the worked material during use. Yet, other processes during the life cycle of a stone tool or after deposition may leave residues and these residues may potentially lead to misinterpretations. We present a blind test that was designed to examine this issue. Results confirm that production, retouch, prehension, hafting, various incidental contacts during use and deposition may lead to residue depositions that significantly affect the accurateness of identifications of tool-use. All currently applied residue approaches are concerned. We therefore argue for a closer interaction with independent wear studies and a step-wise procedure in which a low magnification of wear traces is used as a first step for selecting potentially used flakes in archaeological contexts. In addition, residue concentrations on a tool’s edge should be sufficiently dense before linking them with use.

## Introduction

Since its introduction in the late nineteen seventies [1,2], analysis of residue on stone artefacts has gradually developed into a valuable procedure to obtain often unique data on tool function and the use of plants and animals, otherwise invisible in the archaeological record [3,4]. Today, residue analysis is more widely applied and has resulted in hypothesis evaluation and innovative interpretations [5–9], in particular for the Middle Palaeolithic and Middle Stone Age [10–13]. Its validity has been examined through blind testing [14–17], and progress has been made to improve its identifications through various procedures [18,19]. Much of this progress has focussed on improving the taxonomic resolution and confidence level of residue identifications, which are essential elements of a reliable residue analysis. We believe, however, that there is another important yet unresolved problem that relates to understanding the process of residue deposition, including residues that are not directly related to tool-use.

Several classes of residue (e.g. wood fibres and starch) observed on flaked stone tools are assumed to be a direct effect of edge-use for craft or food processing tasks, while other residues, notably gum and resin traces are selectively interpreted as hafting media. However, we still have little understanding of the diverse processes that lead to residue deposition. Based on ethnoarchaeological analyses, we know that plant materials unrelated to use, in particular starch, potentially mix with and contaminate residue traces that are related to use [20]. It is likely that many other processes may lead to residue deposition in the same way that we know wear formation is complex. For example, it has been argued that tool production and retouch [21,22], prehension [23] and hafting [24] and weathering may lead to wear formation, and criteria have been proposed for distinguishing these classes of wear [25]. However, for residues, we currently do not yet have such comprehensive reference criteria to distinguish actual tool-use from other origins, even though it is often argued that the distribution, smearing and directional patterning of residues demonstrate use or a particular functional aspect [1,16,20,26–32]. While these may be valid arguments, there is usually little control over the extent to which residues from various other processes (during or after a stone tool’s life cycle) may create confusion or lead to misinterpretation. We propose a blind test as an ideal way to examine this issue. While previous blind tests have focussed on the correct identification of use residues [14–16], we designed a blind test to examine which processes may lead to residue deposition and to what extent such residues may cause interpretative confusion and hinder correct identifications about whether a stone tool was used and how it was used. In addition, given the significant development of residue analysis over the past years, various analytical procedures are now applied to characterise tool residues and we have, as yet, little comparative data to evaluate their potential. A second aim of our blind test was, therefore, an evaluation of two current approaches, each of which has emerged historically in different regions.

## Background

### Residue analysis

Stone tool residue analysis has its origins in North America (e.g., [1,2,33–35]) but developed a strong research tradition in Australia, following thesis research in the early 1980s by Fullagar [36], and after Tom Loy moved to Australia in 1987 [37]. Much of the earlier research focussed on microscopically distinct structures such as starch grains, phytoliths and blood cells (e.g., [3,4,38–43]). However, Loy in particular promoted molecular, biochemical and genetic analyses, and his students continued these studies (see references in [37]).

The residue analyses performed in Australia generally include an on-tool screening (hereafter referred to as in situ analysis, [44], followed by removal of residues from selected areas for further study [8,9,40]. Generally, identifications rely on transmitted-light observations of extracted residues, as analysts consider residues to often lack distinctly visible structures and to be insufficiently diagnostic under reflected-light (e.g., [45]). Residue extraction is currently not common practice in Europe and South Africa [46]. In the latter regions, an in situ analysis is more common, with a detailed registration of all residue locations on the tool surface (e.g., [10,11,44,47–49]). The emphasis of European and South African approaches is more reliant on the distribution of the residues and analysts do consider residues to be sufficiently distinctive under reflected-light [15,47,49]. The different approaches entail particular microscopes and lighting arrangements: a reflected-light microscope and/or small digital microscope such as a Dino-Lite in Europe (e.g., [10,47]) and South Africa (e.g., [11,12]); and a combined use of reflected- and transmitted-light microscopes in Australia (e.g., see papers in [3,50].

Compound specific stains are commonly used in medicine and biology, but despite early archaeological application of stains, only recently has staining been systematically applied to archaeological artefacts [40,51,52]. Staining, usually of extracted residues, has the advantage of being an easy and rapid way to examine the presence of certain molecular structures (e.g., collagen) by varying the type of stain that is used. Other more specialised analytical procedures, such as scanning electron microscopy with energy dispersive X-ray spectroscopy (SEM-EDX), gas chromatography-mass spectrometry (GC–MS), liquid chromatography-mass spectrometry (LC–MS), Fourier transform infrared spectroscopy (FTIR) and Raman spectroscopy (e.g. [19]), are currently being explored for residue analysis. These methodologies are not considered here but we note current applications to both extracted and in situ residues.

### Blind tests

Several residue blind tests for optical microscopy have been performed in the past. Hardy and Garufi [14] performed a blind test on 50 stone tools. All of these were used on wood, in variable situations, and the goal was to examine whether detailed identifications concerning wood processing would be possible. A series of blind tests including a broader variety of tool uses was performed by Lyn Wadley, Marlize Lombard and Bonny Williamson. Their first blind test [16] included 28 flaked stone tools, most of which were used, but some remained unused, or residues were intentionally applied to them. Their goal was to examine the analyst’s ability to identify stone tool use. Tools were placed in plastic bags immediately after use and none of the flakes were washed before being handed over to the analyst. This test was heavily critiqued by Crowther and Haslam [17] for its protocols and inaccuracies. A second blind test was published by Lombard and Wadley [15] and included an additional set of 26 tools. Again, flakes remained unwashed and the test was mainly focused on the abilities of the analyst to correctly identify tool use.

## Blind Test Methodology

### Aims

The aim of the designed blind test was to contribute to the methodological development of residue analysis by increasing its reliability and understanding its limitations. The test has two specific objectives, detailed below, one related to the reliable interpretation of residue causes and a second related to the methods of analysis.

A first objective of the blind test was to investigate whether residues could result from different life cycle stages of a stone tool (from production up to discard, cf. [24]) or from various deposition scenarios after tool discard, and whether the residues that were perhaps deposited could possibly cause confusion when trying to interpret whether a stone tool was used and how it was used. The goal is to understand the causes of this potential confusion and to develop criteria that can improve the reliability of identifications made on archaeological stone tools thanks to a better understanding of the limitations of current approaches. Therefore, not all experimental flakes were used and a number of used and unused flakes were buried or deposited in various contexts in order to examine how the build-up of incidental residues may affect interpretations of tool use. It has to be stressed that at no instance were incidental or confusing residues strived for and no planting of residues was performed. A special interest concerned the possible confusion between retouch, hafting and use residues, which influences the composition of the experimental tool set. Participants were not informed about the key goal of this blind test and assumed they were involved in a “standard” residue blind test that would focus on the general accuracy of use identifications.

A second objective of the blind test was to evaluate potential limitations and advantages of two commonly used approaches in residue analysis in order to identify how residue analysis—given its time-intensive nature—could be most efficiently integrated into studies on stone tool use (in the broadest sense). Similar to previous blind tests, the methods under consideration are restricted to optical microscopy only, but in contrast to previous tests, transmitted-light microscopy was also included (next to reflected-light microscopy). In order to allow an evaluation of both approaches, a phased procedure was opted for with a separate interpretation per included analytical protocol. As part of the second analytical phase, staining could also be applied.

The test involved three analysts but was not designed to evaluate individual capacity to identify residues. Although the evaluation of each analyst’s identification skills is important, in particular as so few residue blind tests have yet been performed, we believe that it is also important at this stage of methodological development to reflect on how and what residues stick to a stone tool’s surface, how these adhesive properties may influence functional interpretations, and how possible misinterpretation of tool use can be prevented in the future.

The focus of the blind test is on residues and not on wear traces. It is intended to examine the potential and difficulties of residue analysis in its own right and not (1) as part of an integrated approach in which also some wear traces are considered (e.g., [11,47,48,53,54]), (2) as a specific small case study in addition to use-wear analysis (e.g., [55]), (3) as an approach in which residue and wear analyses are combined as specific methodologies in a phased procedure, generally with one analyst (e.g., [56]), or (4) as independent methodologies by two separate analysts (e.g., [20,56]. It is important to stress the difference between an integrated approach and a phased or independent approach: they are likely to be equally adequate, but they differ in strategy. In an integrated approach, one analyst uses use-wear evidence in support of residue interpretations (or more rarely, residues in support of use-wear). It generally involves the examination of some wear features only (in particular edge damage, but also rounding) because other wear features (polish, striations—i.e., not smearing) can only be adequately assessed after cleaning (and thus removal of residues). In the majority of those studies, artefacts are not cleaned, making it impossible to adequately observe all wear traces. In such cases, the wear and residue evidence constitute two different, but not independent lines of evidence. In an independent approach, a wear analysis is performed after completion of the residue analysis—as cleaning is required—in a sequential procedure with two different analysts. Preferably, results are confronted only after completion of both analyses. A phased procedure takes an intermediate position between both as only one analyst is involved. An integrated and phased approach have been applied most frequently up to now, for instance by analysts like (integrated) Lombard (e.g., [11,44,48]), Hardy (e.g., [10,47,57]), and Robertson (e.g., [54,58]), and (phased) Fullagar [59] and Kononenko [60]. We agree with these analysts that residue analysis is best combined with a wear analysis, either in an integrated, a phased or in an independent way, to reliably address questions of tool use (in a broad sense). The designed blind test is not a test of these different ways of combining both, as it is often more a question of practical possibilities (raw material, preservation, analyst availability, etc.) and preferences, than of accuracy. We nevertheless hope that the blind test may perhaps provide some useful directions in terms of how residue analysis, with its different existing methodologies, and wear analysis may be most efficiently integrated in a sequential approach to reliably address functional questions in future studies.

### Experimental protocol

According to the objectives of this test, the experimenter did not wear gloves during production or use and did not attempt to create laboratory-clean experimental conditions. On the contrary, experimental conditions—from tool production up to the transfer of the stone tool to a plastic bag—were designed to simulate possible prehistoric situations of residue transfer and activities were performed outside. The idea was to allow the incidental deposition of residues during use or handling, but no residues were planted or deliberately attached to confuse analysts.

Knapping and retouch were performed with various hammers, both mineral and organic, in order to examine whether residues deposited by bone, antler or wooden hammers or retouchers could potentially be mistaken for use-related residues. Both hand-held and hafted artefacts were included. Only part of the tools was used; tool use varied and involved different animal materials, hard and soft, and different plant materials including wood. Use motions varied (e.g., scraping, cutting, grooving, perforating) and use durations ranged from 20 minutes up to one hour. Both retouched and unretouched artefacts were used to prevent use-related assumptions based on general stone tool morphology. Some artefacts and used tools were also deposited outside or buried.

### Experimental data

Thirty experimental flint artefacts were included in the blind test (Table 1). All blanks were prepared by Christian Lepers (CL), an experienced knapper and stone tool user, according to guidelines provided by Veerle Rots (VR). Less than half of the blanks were retouched.

**Table 1:** Experimental details on the blind test artefacts (tool use, prehensile mode, blank, cleaning protocol).

Tool Nr	Experimental details	Production	Retouch	Used edge	Use duration	Hand-held / Hafted	Hafting Method	Tool Type	Cleaning
BT1	Freshly knapped; unused	direct percussion, stone	-	none	-	-	-	blade	-
BT2	Hafted and de-hafted; unused	direct percussion, antler	-	none	-	hafted	wrapping with vegetal bindings	blade	-
BT3	Processing fish	direct percussion, antler	-	left and right edge	20'	hand-held	-	blade	running water
BT4	Harvesting cereals	direct percussion, antler	-	left edge	20'	hafted	male, wooden haft + resin	blade	running water + ethanol
BT5	Rolled in hide (2 weeks) and unrolled; unused	direct percussion, sandstone	sandstone	none	-	-	-	endscraper	-
BT6	Freshly knapped and retouched; unused	direct percussion, antler	antler	none	-	-	-	burin	-
BT7	Lying around while resin hafting; unused	direct percussion, antler	-	none	-	-	-	blade	-
BT8	Freshly knapped and retouched; unused	direct percussion, wood (buxus)	antler	none	-	-	-	endscraper	-
BT9	Wood scraping	direct percussion, antler	sandstone	scraper-head + right edge	45'	hafted	juxtaposed, wooden haft + leather bindings	endscraper	running water + ethanol
BT10	Hide cutting	direct percussion, antler	-	left edge	30'	hand-held	-	blade	running water
BT11	Scraping wood and buried for 2 months	direct percussion, antler	sandstone	scraper-head + left edge	1h	hafted	juxtaposed, wooden haft + leather bindings	endscraper	running water
BT12	Freshly knapped and retouched; unused	direct percussion, stone	stone	none	-	-	-	retouched flake	-
BT13	Butchering	direct percussion, antler	-	left and right edge	1h	hand-held	-	blade	running water
BT14	Hafted and de-hafted; unused	direct percussion	-	none	-	hafted	wrapping with animal bindings	blade	-
BT15	Perforating antler	direct percussion, antler	sandstone	distal tip	25'	hand-held	-	perforator	running water
BT16	Freshly knapped and retouched; unused	direct percussion, antler	wood	none	-	-	-	endscraper	-
BT17	Bone grooving	direct percussion, antler	sandstone	burin tip	30'	hand-held	-	burin	running water
BT18	Scraping inner bark	direct percussion, antler	-	left edge	30'	hand-held	-	blade	running water
BT19	Deposition in a river for 2 months; unused	direct percussion, sandstone	-	none	-	-	-	flake	running water
BT20	Processing tubers	direct percussion, antler	-	left edge	30'	hand-held	-	blade	running water
BT21	Freshly knapped; unused	direct percussion, antler	-	none	-	-	-	blade	-
BT22	Hafted and de-hafted; unused	direct percussion, stone	-	none	-	hafted	wooden haft + resin	blade	running water
BT23	Antler scraping	direct percussion, antler	antler	scraper-head	30'	hafted	juxtaposed, wooden haft + leather bindings	endscraper	running water
BT24	Cutting fresh bone and buried for 2 months	direct percussion, wood (buxus)	-	left and right edge	1h	hand-held	-	blade	running water
BT25	Freshly knapped and dropped on organic-rich soil; unused	direct percussion	-	none	-	-	-	blade	-
BT26	Deposition on humic soil for 2 months; unused	direct percussion, sandstone	-	none	-	-	-	flake	running water
BT27	Freshly knapped; unused	direct percussion, wood (buxus)	-	none	-	-	-	blade	-
BT28	Adzing wood (fresh willow)	direct percussion, antler	antler	scraper-head	1h	hafted	latero-distal/juxtaposed, wooden haft + leather bindings	endscraper	running water + ethanol
BT29	Cutting meat and buried for 2 months	direct percussion, wood (buxus)	-	left and right edge	40'	hand-held	-	blade	running water
BT30	Lying around while preparing string; unused	direct percussion	-	none	-	-	-	blade	-

A total of 14 artefacts were used, 3 of which were buried for 2 months following their use. All these artefacts were gently rinsed under running water to remove obvious macroscopically visible residue (see below), before they were handed over to the analysts. Of the 16 unused artefacts, 3 were deposited in varying conditions (specified in Table 1), 3 were hafted but remained unused, 2 were lying nearby while hafting other tools, 4 were freshly knapped and 4 were freshly knapped and retouched.

The 3 deposited flakes were freshly knapped, placed in their specified individual environments for a given duration, and were then recovered and stored in separate plastic bags. Three flakes were hafted without being used and after a week were simply de-hafted again. The resin-hafted tool was heated to allow de-hafting.

### Cleaning

**Fig 1:**
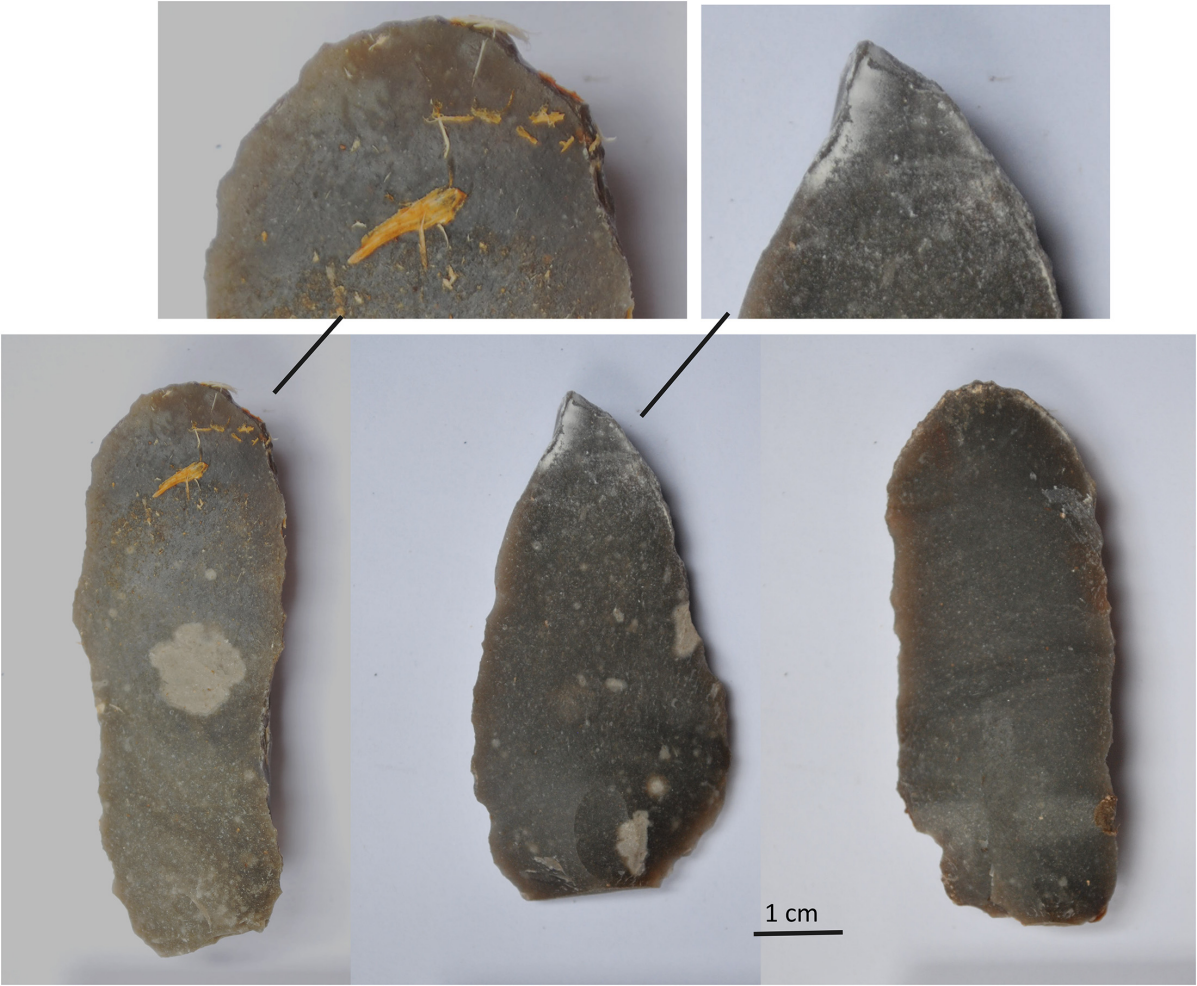
Macroscopically visible residues before washing (from left to right): BT9 (wood scraping), BT17 (bone grooving), BT23 (antler scraping).

In contrast to previous blind tests, used stone tools were gently rinsed under running tap water after use, and air dried for at least one hour before they were handed over to the analysts. This cleaning was considered necessary as a macroscopic examination of the used flakes quickly showed that residues were easily visible with the naked eye (Fig 1). If flakes were left unwashed, no microscope would be required to identify used flakes or even tool use in several cases. Gentle washing has the advantage it ensures the experimental artefact surfaces look similar to the appearance of archaeological stone tools, although it does not physically or biochemically alter or degrade microscopic residues that remain attached. Residue degradation is a factor that is more difficult to reproduce in a blind test setting, but further tests are planned. Unused flakes were also washed under running tap water and air dried. In particular, one experimental flake deposited in a river needed to be washed as green algae were present over the entire stone tool surface. The algae strongly adhered to the stone tool surface and could only be partially removed.

During washing, tools were handled with starch-free gloves and if residue adhesion was strong, stone tools were gently rubbed with gloved fingers while being kept under running water. Macroscopically visible residues (i.e., adhering fibres or organic fragments) were removed at this stage. In the case of 3 tools, some obvious residues (e.g., resin or wood remains) were adhering so strongly that ethanol had to be gently applied to the areas in question (Table 1).

### Analytical protocol

The two analytical approaches that are considered are: (1) the analysis of in situ residues under reflected light microscopy and (2) the analysis of extracted residues under transmitted light microscopy. The analytical protocol included two main stages: first, in situ observation of residues on the stone artefact surfaces; and second, an extraction of selected residues with observations under transmitted light. As an element of the second analytical stage, participants could selectively apply staining. The analyst had to select a suitable stain based on visual clues of a particular residue. It was documented how the stain aided interpretation. As the focus of the blind test concerned residues and not wear traces, participants were not allowed to examine wear traces. It was thus a major advantage that two of the participants had no or moderate experience with wear traces on flaked stone.

Specific forms were used during the blind test on which the (assumed) nature and interpretation of each of the observed residues was listed and its location was recorded on a printed picture of the stone tool. Per analytical phase, an interpretation of the stone tool was provided, including a degree of confidence of this interpretation based on a scale from 0 (uncertain), 1 (poor confidence), 2 (moderate confidence), 3 (high confidence), to 4 (certain). There was no set time frame and analysts could invest as much time in the analysis as they considered necessary. Two participants followed the designed protocol (Analyst 1 and 2). A third analyst only performed a quick screening by combining a stereoscopic microscope (magnifications up to 56x) and a metallurgical reflected-light microscope (magnifications up to 500x), investing about 20–30 minutes maximum per tool. This analyst only provided one interpretation per tool.

There was no communication of information between the analysts. Analyst 1 and 2 independently performed the in situ observation. Subsequently, they decided where to take extractions and they prepared their own slides based on the extracted solutions. Analyst 3 examined the blind test flakes after Analyst 1 and 2 had completed the full test and without being informed of their results.

#### Phase 1

**Fig 2:**
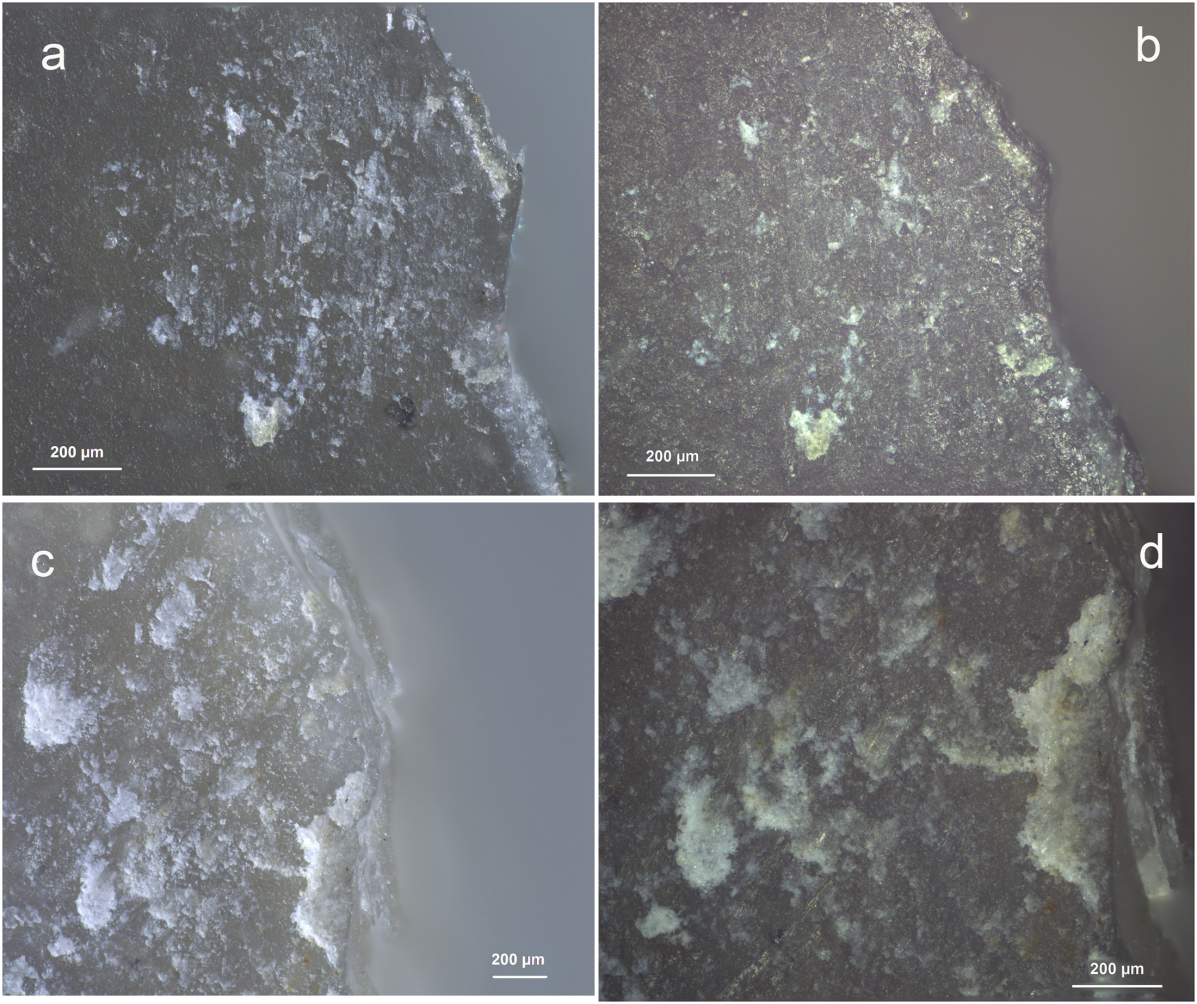
comparison of residue appearance under V16 Zeiss zoom microscope with magnifications up to 180x (a & c) and reflected light metallurgical microscope (Zeiss AxioImager) with magnifications up to 500x: b &d) images from the same residues. **a & b)** wood from retouching; **c & d)** antler from retouching (a: 160x; b, c, d: 100x).

The two main participants of the test (Analyst 1 and 2) first performed an in situ screening of the stone tools. All available microscopes could be used, including a binocular stereomicroscope (magnifications up to 56x), a Zeiss V16 motorised AxioZoom microscope (magnifications up to 180x) and a Zeiss or Olympus metallurgical reflected-light microscope (magnifications up to 500x). Both analysts chose to preferentially use the AxioZoom microscope given its easy use and the good contrast it provides (Fig 2). It was nevertheless frequently combined with the metallurgical reflected-light microscope. Residues were described and interpreted, their location was recorded, and a first interpretation of the stone tool was provided. Photographs were captured with a Zeiss AxioCam ICc5, permitting both colour and black and white digital images (recorded as TIFF files).

#### Phase 2

Subsequently, residues were examined under high magnification with reflected light to identify relevant areas for residue extraction. Residues were extracted using an adjustable pipette fitted with disposable polypropylene tips. Up to 50 μl of distilled water was applied to the desired area and gently agitated with the pipette tip before being removed and transferred to clean glass slides (wiped with ethanol). The advantage of pipette extractions (as opposed to ultrasonication which requires larger portions of the artefact to be submerged in solution) is that the analyst may target a particular residue and/or residue location, while leaving much of the remaining in situ residues intact on the tool surface. The link with the distribution pattern on the stone tool is thus maintained. Glass slides were examined under transmitted light with the aid of a metallurgical microscope (Zeiss Axioscope or Olympus BH2) with objective lenses 50x, 100x, 200x, 400/500x, 1000x, cross-polarising filters and DIC. Photographs of constituent residue material were captured with an Olympus Infinity 2 camera or Zeiss AxioCam ICc5, permitting both colour and black and white digital images (recorded as JPEG or TIFF files). An interpretation was provided based on the extracted residues.

Following microscopic analysis of extracted residues, several samples were selected for staining so that any highly degraded, fragmented or amorphous residues could be identified. Methylene Blue (C_16_H_18_N_3_SCI) was used to highlight non-lignified cell walls such as cellulose fibres within plant material. The stain, which is a water soluble dye, binds to the acidic pectins on the cellulose cell wall that become stained with various shades of blue [61–63]. Orange G (C_16_H_10_N_2_Na_2_O_7_S_2_)–an acidophilic dye, was used to identify animal fibres such as collagen and keratin by binding with proteins in the target material, typically staining them orange [62,64]. Approximately 5 μl of the nominated stain was applied to selected residue mixtures and left for ~10–20 minutes to allow the stain to develop. Excess stain was then rinsed from the glass slide before it was again examined under transmitted light.

The presence of haemoglobin (and other iron containing materials) was assessed using the presumptive haemoglobin specific chemical reagent test strip (Hb-CRTS): Siemens Hemastix^®^ test strips [65]. Tools that were presumed to be in contact with animal material (as implied by the presence of collagen fibres or the visual appearance of other tool-use residues) were subjected to Hemastix^®^ testing. Five micro-litres of solution from the water-extracted residue sample was placed on the Hemastix^®^ test pad and left for 1 minute to see if a colour change occurred. If colour change had not occurred after this time, the sample was deemed negative for haemoglobin. Evaluations of colour change were assessed within 1 minute, after which the pad can auto-oxidise and change colour, creating a false-positive result. Colour change was ranked on a scale of 0–5 as recommended on the Hemastix package: 0 representing no change in colour; 1–2 for a speckled colour change and 3–5 for a broad colour change ranked on increasing darkness. These rankings correspond to negative, slight trace, trace, small, moderate, and large traces of haemoglobin, respectively.

Analyst 1 chose to make use of the two stains; while Analyst 2 chose only to make use of Orange G. Analyst 3 did not apply any stains as the analysis was limited to a general in situ screening of the stone artefacts. This blind test was not designed for examining the accuracy or specificity of the stains themselves; their validity and limitations have been established in modern biological and archaeological applications [52,65]. The question here was whether these stains would aid in correctly assessing whether an artefact was used and what the artefact was used for.

## Results

As the first objective of the blind test was to evaluate whether residues were deposited during the different life cycle stages and whether these could cause confusion, we discuss the results by focusing on correct identification of: (1) used and unused flakes; (2) tool-use and used edges; and (3) prehensile mode. This procedure allowed us to identify which residues were acquired from use and which were acquired through incidental contact with other materials during the various phases of the stone tool life cycle, such as manufacture, discard and handling, and various depositional contexts that were erroneously linked with use.

In order to allow a comparison between the different analytical protocols and their success rates, results are divided per analytical level. Results from the in situ observations of Analyst 1 and 2 are summarised in Table 2 and their results from looking at extracted residues under transmitted light are summarised in Table 3. For Analyst 3, only one level of results is provided given that only a general screening was performed. These are summarised in Table 4.

**Table 2:** Interpretations of Analyst 1 and 2 based on in situ analysis of residues, directly on the stone artefacts (BT: blind test; CL: confidence level—on scale from 0 = uncertain to 4 = certain). Scores: 0 (wrong), 0,5 (partially correct), 1 (correct).

BT Nr	Analyst 1 / In-situ observation													Analyst 2 / In-situ observation												
	Used vs unused	CL	*Score Use*	Tool Use	CL	*Score / worked material*	*Score / motion*	Use location	CL	*Score location*	Prehensile mode	CL	*Score / hafting*	Used vs unused	CL	*Score Use*	Tool Use	CL	*Score / worked material*	*Score / motion*	Use location	CL	*Score location*	Prehensile mode	CL	*Score / hafting*
BT1	used	1	***0***	wood scraping	0–1	***0***	***0***	left edge	1	***0***	hand-held	0	***0***	unused	1	***1***	unused	1	***1***	***1***	none	-	***1***	none	-	***1***
BT2	used	1	***0***	cutting animal material	1	***0***	***0***	left edge	1	***0***	hafted	1	***1***	unknown	0	-	unknown	0	-	-	unknown	-	-	unknown	-	-
BT3	used	4	***1***	slicing grasses	3–4	***0***	***1***	right ege	4	***0*,*5***	hafted	2	***0***	unknown	2	-	unknown	2	-	-	residues all over	0	-	unknown	1	-
BT4	unused	2	***0***	unused	2	***0***	***0***	none	2	***0***	hafted	4	***1***	used	2	***1***	plant processing	2	***1***	***1***	residues all over	0	-	hand-held	2	***0***
BT5	used	4	***0***	scraping antler	2–3	***0***	***0***	left edge	2–3	***0***	hand-held	0	***0***	used	1	***0***	animal processing	1	***0***	***0***	scraper-head	4	***0***	hafted	3	***0***
BT6	used	3	***0***	scraping antler	3	***0***	***0***	proximal left	3	***0***	0	0	***0***	unknown	0	-	unknown	0	-	-	unknown	-	-	unknown	-	-
BT7	unused	3	***1***	unused	3	***1***	***1***	none	3	***1***	none	3	***1***	used	3	***0***	cutting wood	3	***0***	***0***	unknown	-	-	hand-held	2	***0***
BT8	used	4	***0***	scraping meat from bone	3	***0***	***0***	distal scraper-head	4	***0***	hand-held	3	***0***	used	4	***0***	animal processing	4	***0***	***0***	scraper-head	4	***0***	hafted	3	***0***
BT9	used	4	***1***	scraping wood	3	***1***	***1***	distal edges	0	***1***	?	0	***0***	used	4	***1***	scraping wood	4	***1***	***1***	scraper-head	3	***1***	hafted	2	***1***
BT10	used	4	***1***	slicing/cutting organic material/meat	2	***0***	***1***	both edges	1	***0*,*5***	hafted	1	***0***	used	3	***1***	cutting wood	3	***0***	***1***	right edge	2	***0***	hafted	2	***0***
BT11	used	1	***1***	scraping wood (earth, buried?)	1	***1***	***1***	distal	1	***1***	possibly hafted	0	***0*,*5***	used	2	***1***	scraping skin	2	***0***	***1***	scraper-head	3	***1***	hafted	2	***1***
BT12	unused	3	***1***	unused	3	***1***	***1***	none	3	***1***	none	3	***1***	unknown	1	-	unknown	1	-	-	unknown	2	-	unknown	1	-
BT13	used	3	***1***	butchering	3	***1***	***1***	right ege	3	***0*,*5***	hafted	2	***0***	used	3	***1***	scraping skin	3	***0*,*5***	***0***	residues all over	0	-	hand-held	3	***1***
BT14	used	3	***0***	plant, wood	0	***0***	***0***	left edge	2	***0***	?	0	***0***	used	3	***0***	wood cutting	3	***0***	***0***	unknown	0	-	hand-held	2	***0***
BT15	unused	0	***0***	unused	0	***0***	***0***	none	0	***0***	none	0	***0***	unused	4	***0***	unused	4	***0***	***0***	none	0	***0***	none	2	***0***
BT16	used	3	***0***	scraping antler or bone	3	***0***	***0***	right edge	3	***0***	hand-held	3	***0***	used	0	***0***	wood scraping	0	***0***	***0***	unknown	0	-	hafted	0	***0***
BT17	used	3	***1***	bone	3	***1***	***0***	left edge	4	***0***	hafted	2	***0***	used	2	***1***	bone processing	2	***1***	***0***	unknown	2	-	hafted	1	***0***
BT18	used	4	***1***	sawing wood	3	***1***	***0***	left edge	4	***1***	hand-held	3	***1***	unused	2	***0***	buried in sediment	2	***0***	***0***	none	0	***0***	hafted	2	***0***
BT19	used	4	***0***	cutting fish	3	***0***	***0***	left edge	3	***0***	hand-held	1	***0***	used	3	***0***	cutting meat and the buried or left on the ground (butchering)	3	***0***	***0***	unknown	0	-	hand-held	2	***0***
BT20	used	2	***1***	plant working	2	***1***	***1***	right edge	1	***0***	hafted	1	***0***	used	3	***1***	cutting plants	3	***1***	***1***	residues all over	0	-	hand-held	2	***0***
BT21	used	2	***0***	cutting/slicing plant	1	***0***	***0***	right edge	1	***0***	hand-held	1	***0***	used	0	***0***	cutting dry bone or antler	0	***0***	***0***	unknown	0	-	hand-held	0	***0***
BT22	used	1	***0***	unsure	0	***0***	***0***	distal	1	***0***	hafted	1	***1***	unused	1	***1***	only haft related residues	1	***1***	***1***	unknown	-	-	hafted	2	***1***
BT23	used	4	***1***	bone/antler scraping	4	***1***	***1***	distal	4	***1***	hafted	4	***1***	used	2	***1***	animal processing (butchering)	2	***0***	***0***	scraper-head	4	***1***	hand-held	3	***0***
BT24	used	2	***1***	digging earth	0	***0***	***0***	right edge	2	***0*,*5***	hand-held	2	***1***	used	3	***1***	animal cutting and then buried	3	***1***	***0***	residues all over	0	-	hand-held	3	***1***
BT25	uncertain	0	-	uncertain	0	-	-	uncertain	0	-	uncertain	0	-	used	1	***0***	cutting dry bone or dry antler	1	***0***	***0***	unknown	0	-	hand-held	2	***0***
BT26	used	1	***0***	process organic material	2	***0***	***0***	distal left edge	1	***0***	hand-held	2	***0***	unused	1	***1***	unused	1	***1***	***1***	residues all over	0	***1***	none	-	***1***
BT27	unused	4	***1***	none	4	***1***	***1***	none		***1***	none	4	***1***	unused	2	***1***	unused	2	***1***	***1***	none	-	***1***	hafted	2	***0***
BT28	used	4	***1***	wood scraping	4	***1***	***0*,*5***	distal scraper-head	4	***1***	hafted	2	***1***	used	3	***1***	scraping wood	3	***1***	***0*,*5***	scraper-head	3	***1***	hafted	3	***1***
BT29	used	1	***1***	digging sediment	0	***0***	***0***	left edge	1	***0*,*5***	hand-held	1	***1***	perhaps used	1	***0*,*5***	buried or used for scraping stone	2	***0***	***0***	unknown	-	-	unknown	-	-
BT30	uncertain	0	-	uncertain	0	-	-	uncertain	0	-	uncertain	0	-	unknown	0	-	unknown	0	-	-	unknown	-	-	hafted	2	***0***

**Table 3:** Interpretations of Analyst 1 and 2 based on an analysis of extracted residues under transmitted light. Staining was applied when considered relevant by the analyst (BT: blind test; CL: confidence level—on scale from 0 = uncertain to 4 = certain). Scores: 0 (wrong), 0,5 (partially correct), 1 (correct).

BT Nr	Analyst 1													Analyst 2												
	Transmitted light										Staining			Transmitted light										Staining		
	Used vs unused	CL	Score Use	Tool Use	CL	Score / worked material	Score / motion	Prehensile mode	CL	Score / prehensile mode	Stain (Orange G, Methylene Blue & Hemastix)	Reaction on use or hafting residue?	Did stain affect interpretation?	Used vs unused	CL	Score Use	Tool use	CL	Score worked material	Score motion	Prehensile mode	CL	Score / prehensile mdoe	Stain (Orange G)	Reaction on use or hafting residue?	Did stain affect interpretation?
BT1	unused	3	**1**	unused	1	**1**	**1**	none	3	**1**	MB positive but contamination because fragmented	0	confirms contamination	unused	1	**1**	unused	1	**1**	**1**	none	1	***1***	-	-	-
BT2	used	1	**0**	cutting animal	2	**0**	**0**	hafted	3	**1**	OG—few fragments positive	0	negative influence	unknown	0	-	unknown	0	-	-	unknown	-	-	Negative	1	uncertainty maintained
BT3	used	4	**1**	slicing fish	3	**1**	**1**	hand-held	3	**1**	OG—confirms collagen	1	confirm	unknown	2	-	unknown	2	-	-	unknown	-	-	-	-	-
BT4	uncertain	0	-	uncertain	1	-	-	hafted	4	**1**	OG—negative, MB—positive	1	uncertainty maintained	used	3	**1**	Plant processing	3	**1**	**1**	hand-held	2	***0***	-	-	-
BT5	used	2	**0**	antler scraping	2	**0**	**0**	hand-held	2	**0**	OG—negative	1	contradiction, but interpretation maintained	used	4	**0**	Animal processing	4	**0**	**0**	hafted	3	***0***	Positive	0	negative influence
BT6	used	3	**0**	antler scraping	3	**0**	**0**	hand-held	3	**0**	OG—positive; MB—positive	0	negative influence	unknown	0	-	unknown	0	-	-	unknown	-	-	Negative	1	uncertainty maintained
BT7	unused	3	**1**	unused	3	**1**	**1**	none	3	**1**		-	-	used	1	**0**	cutting wood	1	**0**	**0**	hand-held	2	***0***	-	-	-
BT8	used	4	**0**	scraping meat from bone	3	**0**	**0**	hand-held	4	**0**	OG—positive	0	negative influence	used	4	**0**	Animal processing	4	**0**	**0**	hafted	4	***0***	-	-	-
BT9	used	4	**1**	wood scraping	4	**1**	**1**	hand-held	1	**0**	MB—positive	1	confirm	used	4	**1**	scraping wood	4	**1**	**1**	hafted	4	***1***	-	-	-
BT10	used	4	**1**	slicing meat	3	**0**	**1**	hand-held	3	**1**	OG—positive	1	confirm	used	3	**1**	cutting wood	3	**0**	**1**	hafted	3	***0***	-	-	-
BT11	used	4	**1**	scraping wood	2	**1**	**1**	hafted	1	**1**	MB—positive; OG—negative	1	confirm	used	4	**1**	scraping skin	4	**1**	**0**	hafted	4	***1***	Positive	0	negative influence
BT12	unused	4	**1**	unused	4	**1**	**1**	none	4	**1**	-	-	-	unknown	1	-	unknown	1	-	-	unknown	-	-	Positive (only stained little parts)	0	uncertainty maintained
BT13	used	4	**1**	butchering	4	**1**	**1**	hafted	1	**0**	-	-	-	used	4	**1**	scraping skin	4	**0,5**	**0**	hand-held	4	***1***	-	-	-
BT14	used	3	**0**	plant processing	1	**0**	**0**	hand-held	1	**0**	MB—positive (but uncommon)	0	negative influence	used	3	**0**	wood cutting	3	**0**	**0**	hand-held	1	***0***	-	-	-
BT15	unused	1	**0**	unused	1	**0**	**0**	none	1	**0**	-	-	-	unused	4	**0**	Not used	4	**0**	**0**	none	0	***0***	-	-	-
BT16	used	3	**0**	antler/bone scraping	3	**0**	**0**	hand-held	3	**0**	OG—positive	0	negative influence	used	0	**0**	animal processing	0	**0**	**0**	hand-held	0	***0***	Negative	1	stain ignored
BT17	used	3	**1**	starchy plant processing	3	**0**	**0**	hafted	2	**0**	OG—negative	0	negative influence	unknown	2	-	unknown	2	-	-	hafted	1	***0***	Positive	1	uncertainty maintained
BT18	used	4	**1**	sawing fresh wood	3	**1**	**0**	hand-held	3	**1**	-	-	-	used	2	**1**	plant processing, buried in sediment	2	**1**	**0**	hafted	2	***0***	-	-	-
BT19	used	4	**0**	cutting fish	3	**0**	**0**	hand-held	1	**0**	OG—positive; Hemastix—positive	0	negative influence	used	3	**0**	cutting meat (butchering)	3	**0**	**0**	hand-held	3	***0***	Positive	0	negative influence
BT20	used	3	**1**	plant processing	3	**1**	**1**	hafted	1	**0**	-	-	-	used	4	**1**	cutting plants	4	**1**	**1**	hand-held	1	***0***	Positive	0	ignored
BT21	used	2	**0**	cutting/slicing plant	1	**0**	**0**	hand-held	1	**0**	MB—positive; OG—positive	0	negative influence	unknown	0	-	unknown	0	-	-	hand-held	0	***0***	Postive	0	uncertainty maintained
BT22	used	2	**0**	antler scraping	2	**0**	**0**	hafted	1	**1**	OG & MB—both positive	0,5	negative influence	unknown	1	-	unknown	1	-	-	hafted	1	***1***	Negative	1	uncertainty maintained
BT23	used	4	**1**	bone/antler scraping	3	**1**	**1**	hafted	3	**1**	MB & OG—both positive; Hemastix slight trace of haem	1	confirm	used	2	**1**	animal processing (butchering)	2	**0**	**0**	hand-held	2	***0***	Positive	1	confirm
BT24	used	2	**1**	digging sediment/earth	1	**0**	**0**	hand-held	2	**1**	-	-	-	perhaps used	2	**0,5**	buried and maybe used for animal processing	2	**1**	**0**	hand-held	2	***1***	Positive	1	uncertainty maintained
BT25	uncertain	0	-	uncertain	0	-	-	hafted	1	**0**	-	-	-	used	2	**0**	dry bone or antler	2	**0**	**0**	hand-held	0	***0***	Positive	0	negative influence
BT26	used	1	**0**	uncertain	0	-	-	uncertain	0	-	MB & OG—both positive	0	negative influence	unused	0	**1**	Not used	0	**1**	**1**	none	0	***1***	Negative	1	confirm
BT27	unused	4	**1**	unused	4	**1**	**1**	none	4	**1**	-	-	-	unused	2	**1**	Not used, only haft related	2	**1**	**1**	hafted	2	***0***	-	-	-
BT28	used	4	**1**	wood scraping	1	**1**	**0,5**	hafted	2	**1**	-	-	-	used	3	**1**	scraping wood	3	**1**	**0,5**	hand-held	3	***0***	-	-	-
BT29	uncertain	0	-	inorganic / sediment	0	**0**	**0**	uncertain	0	-	-	-	-	perhaps used	2	**0,5**	buried or used for scraping stone	2	**0**	**0**	unknown	-	-	-	-	-
BT30	uncertain	0	-	uncertain	0	-	-	uncertain	0	-	MB & OG—both positive	0	uncertainty maintained	unknown	1	-	unknown	1	-	-	hafted	1	***0***	Negative	1	uncertainty maintained

**Table 4:** Interpretations of Analyst 3 based on a broad screening of the blind test artefacts. Scores: 0 (wrong), 0,5 (partially correct), 1 (correct).

BT Nr	Used vs unused	*Score*	Tool use	*Score*	Used edge	*Score*	Observed potential use residues	Prehensile mode	*Score*	Observed potential hafting residues
BT1	probably unused	***1***	probably none	***1***	none	***1***	no	none	***1***	no
BT2	probably used	***0***	-	-	-	-	dark smear	possibly hafted	***0***	waxy glob
BT3	used	***1***	cutting fish	***1***	left edge	***0*,*5***	fish scale; greasy film and polish	possibly hafted	***0***	brown blob
BT4	used	***1***	-	-	left edge	***1***	waxy residue smear, potential starch	-	-	woody fibres
BT5	used	***0***	scraping	***0***		-	no residues under low magnification; thick residue films under high magnification; polish white smears	-	-	
BT6	used	***0***	grooving bone/antler	***0***	tip	***0***	white/yellow blobs, smears	-	-	
BT7	unused	***1***	none	***1***	none	***1***	no traces	none	***1***	no
BT8	used	***0***	scraping, possibly bone	***0***	scraper-head	***0***	smeared white greasy tissue, possible red blood cells	-	-	
BT9	used	***1***	scraping wood	***1***	scraper-head	***1***	woody fibres, perpendicular alignment	-	-	
BT10	used	***1***	cutting	***0*,*5***	right edge	***0***		hand-held	***1***	
BT11	used	***1***	wood	***0*,*5***	scraper-head	***1***		-	-	
BT12	probably used	***0***	-	***0***	scraper-head	***0***	no distinct traces	-	-	
BT13	used	***1***	cutting; plant	***0*,*5***	both edges	***1***	fibres cf plant + cf hair; possible plant cellulose	hand-held	***1***	
BT14	used	***0***	animal	***0***	proximal end	***0***	fleshy residues	-	-	
BT15	perhaps used	***0*,*5***	-	-	tip	***1***	no macro residues; greasy film	-	-	
BT16	probably used	***0***	-	-	-	-		-		
BT17	used	***1***	-	-	-	-		-	-	
BT18	used	***1***	cutting leather/hide	***0***	-	-		-	-	
BT19	used	***0***	-	-	-	-		hand-held	***0***	
BT20	used	***1***	-	-	-	-		-	-	
BT21	uncertain	-	-	-	-	-		-		
BT22	used	***0***	-	-	-	-		hafted	***1***	
BT23	used	***1***	-	-	-	-		-	-	
BT24	used	***1***	-	-	-	-		-	-	
BT25	unused at LP, possibly used at HP	***1***	-	-	-	-		-		
BT26	possibly used	***0*,*5***	-	-	-	-		no hafting	***1***	
BT27	unused	***1***	-	-	-	-		-		
BT28	used	***1***	wood adzing	***1***	-	-		hafted	***1***	
BT29	probably used	***1***	-	-	-	-		-	-	
BT30	used	***0***	scraping, bone or skin	***0***	-	-	-	hand-held	***0***	

### Used and unused

#### Accuracy

The analysts were able to distinguish between the used (N: 14) and unused flakes (N: 16) in about 50% of the cases (min. 47%—max. 60%; Tables 5 and 6). These accuracy rates may appear to be low, but when the data are examined in closer detail it is clear that the used flakes did not cause much confusion (between 86–100% were correctly identified). Flakes that caused confusion were unused, some of which accumulated residues transferred from their production, from their specific depositional environments, and/or from incidental handling of other experimental materials. These data suggest that residue analysis may be well suited for identifying with relative certainty those flakes that were used, but may overestimate the frequency of used flakes, because incidental residues may be erroneously linked with use. Out of the 14 used flakes, 11 and 14 flakes were correctly identified by the analysts as having been used. Out of the 16 unused flakes, only 3 and 4 flakes were correctly identified as not having been used. Such results highlight the problematic nature of identifying tool residues and support the hypothesis that formed the basis of this blind test: that residues observed on a stone tool are often too quickly linked with use, and that the various other causes of residue accumulation are largely ignored. This is further confirmed by the analysis of hafted and unused flakes. The observation of potential hafting residue led analysts to assume that the flake was also used. Only one correct interpretation by one analyst was obtained for this set of artefacts based solely on in situ observations, while this interpretation was erroneously changed following further study under transmitted-light.

**Table 5:** Summary of results based on in situ observation (up to 180x) for the distinction between used and unused pieces; per category of artefacts (percentages are calculated based on identified pieces).

USED VS UNUSED	Nr of flakes	Analyst 1			Analyst 2			Analyst 3 / screening		
		correct	wrong	not identif	correct	wrong	not identif	correct	wrong	not identif
Used & handheld	**7**	6	1	0	4	2	1	7	0	0
Used & hafted	**4**	3	1	0	4	0	0	4	0	0
Used & buried	**3**	3	0	0	3	0	0	3	0	0
Deposited on material	**3**	0	3	0	1	2	0	0	3	0
Hafted & unused	**3**	0	3	0	1	1	1	0	3	0
Lying around when hafting	**2**	1	0	1	0	1	1	1	1	0
Freshly knapped & unretouched	**4**	1	2	1	2	2	0	3	0	1
Freshly knapped & retouched	**4**	1	3	0	0	2	2	0	4	0
**TOTAL**	**30**	**15**	**13**	**2**	**15**	**10**	**5**	**18**	**11**	**1**
***%***		***50*,*0***	***43*,*3***	***6*,*7***	***50*,*0***	***33*,*3***	***16*,*7***	***60*,*0***	***36*,*7***	***3*,*3***

**Table 6:** Summary of results based on transmitted-light observations for the distinction between used and unused artefacts; per category of artefacts.

USED VS UNUSED	Nr of flakes	Analyst 1			Analyst 2		
		correct	wrong	not identif	correct	wrong	not identif
Used & handheld	**7**	6	1	0	4	1	2
Used & hafted	**4**	3	0	1	4	0	0
Used & buried	**3**	2	0	1	3	0	0
Deposited on material	**3**	0	3	0	1	2	0
Hafted & unused	**3**	0	3	0	0	1	2
Lying around when hafting	**2**	1	0	1	0	1	1
Freshly knapped & unretouched	**4**	2	1	1	2	1	1
Freshly knapped & retouched	**4**	1	3	0	0	2	2
**TOTAL**	**30**	**15**	**11**	**4**	**14**	**8**	**8**
***%***		***50*,*0***	***36*,*7***	***13*,*3***	***46*,*7***	***26*,*7***	***26*,*7***

**Fig 3:**
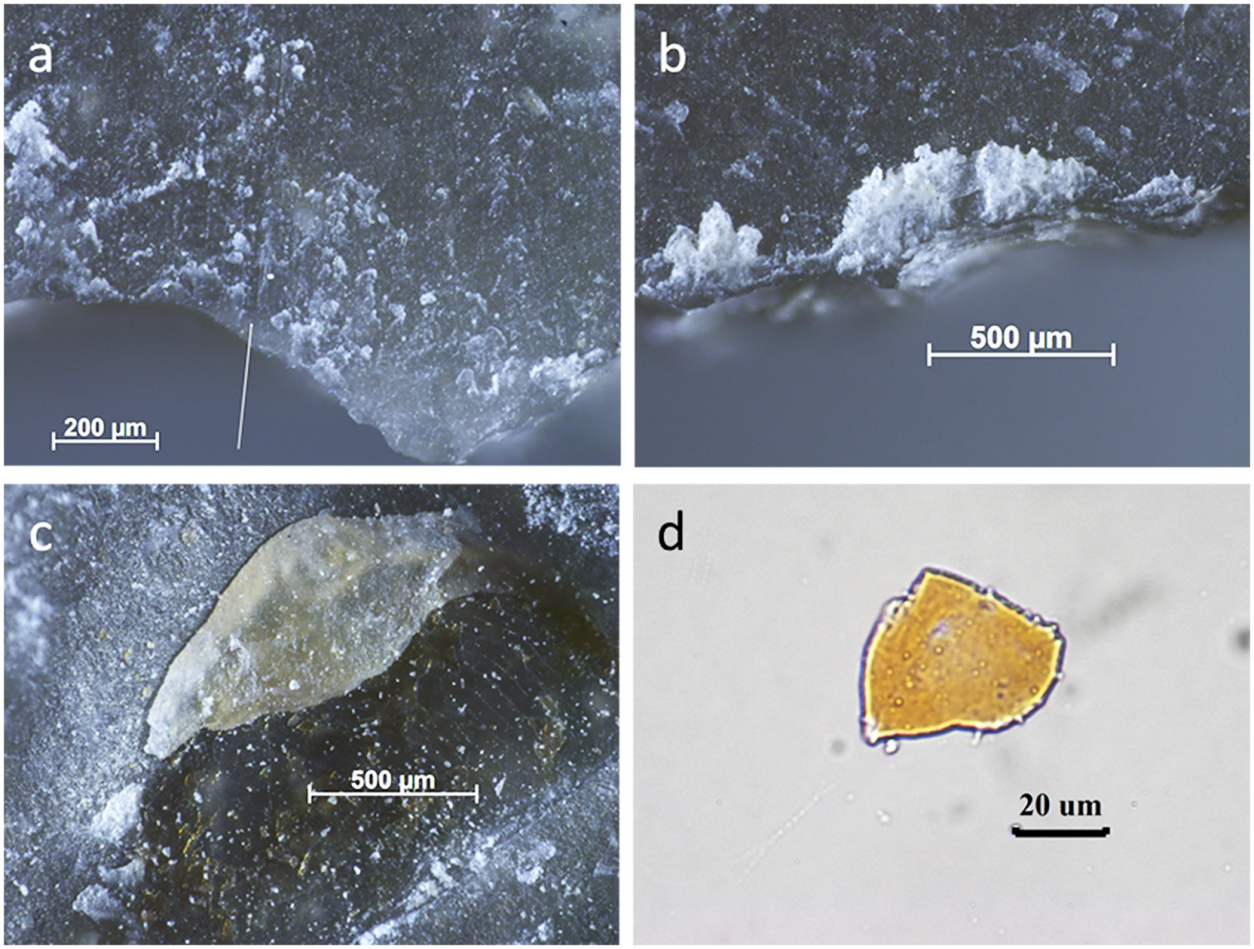
Residues identified on BT6 (unused) as described during blind test. **a)** White use-residue with evident directionality (indicated by arrow) along Edge A of the ventral surface; **b)** high magnification image of white, amorphous residue on tool edge; **c)** possible fatty deposits (beneath scale bar); **d)** collagen residue stained with Orange G, as viewed under transmitted light.

Production and retouch activities proved to be a major cause of residue deposition on the stone tools and these were frequently mistaken for use residues. An example is BT6 (Fig 3), a burin knapped with antler that remained unused (Table 1). It was interpreted as having been used by two analysts and it remained unidentified by a third. Use was inferred based on the presence of smeared white amorphous residue with clear directionality on the tool’s edge. In addition, fatty deposits were observed as well as collagen. The collagen was stained with Orange G by Analyst 1, supporting its identification. This case nicely proves a point: the residues on this flake were all correctly identified and are indeed present on the stone tool surface; however, they were not due to use but due to production. The use of an antler hammer caused directional residues and these can be mistaken for use residues.

Retouch proved to be a major cause of confusion: only one correct interpretation was provided by one analyst for the category of freshly knapped and retouched flakes (N: 4), corresponding to 1/12 (4 artefacts x 3 analysts), in contrast to 6/12 for the unretouched flakes (see Table 5). Indeed, retouch may result in residues that are smeared in a directional pattern on the stone edge; directionality and smearing are arguments that are frequently used to support the use-related origin of residues [18,20,27,66]. Care needs to be taken as these residues may be mistakenly interpreted as being the result of scraping activities especially in the case of organic hammers. The only flake correctly identified as unused among the retouched artefacts was BT12, a flake knapped and retouched with stone. Only Analyst 3 inferred probable use, in spite of specifically noting an absence of convincing traces.

**Fig 4:**
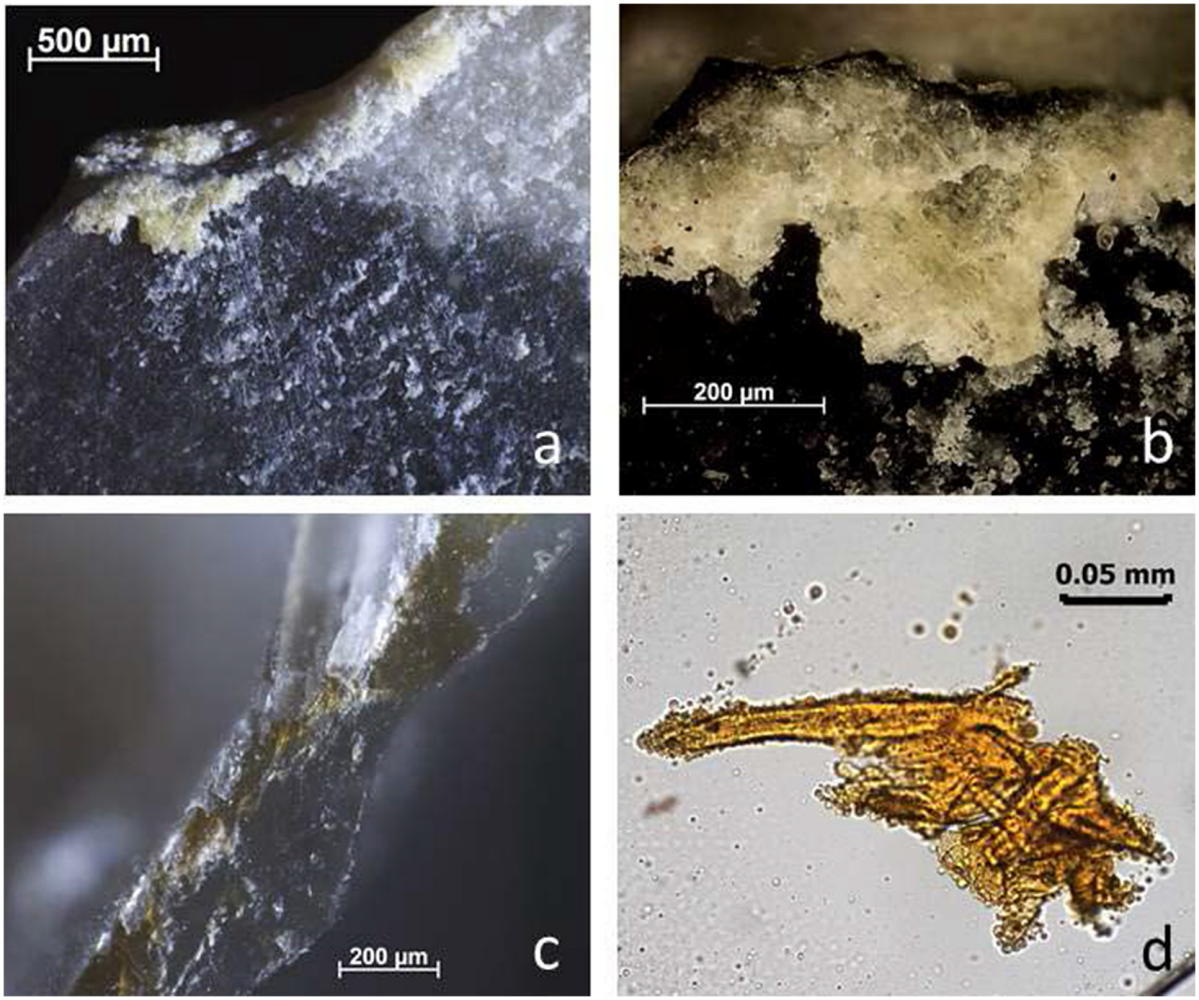
Residues identified on BT16 (unused) as described during blind test. **a-b)** White use-residue with evident directionality (diagonal from edge); **c)** unidentified residue film; **d)** collagen residues stained with Orange G, transmitted light.

**Fig 5:**
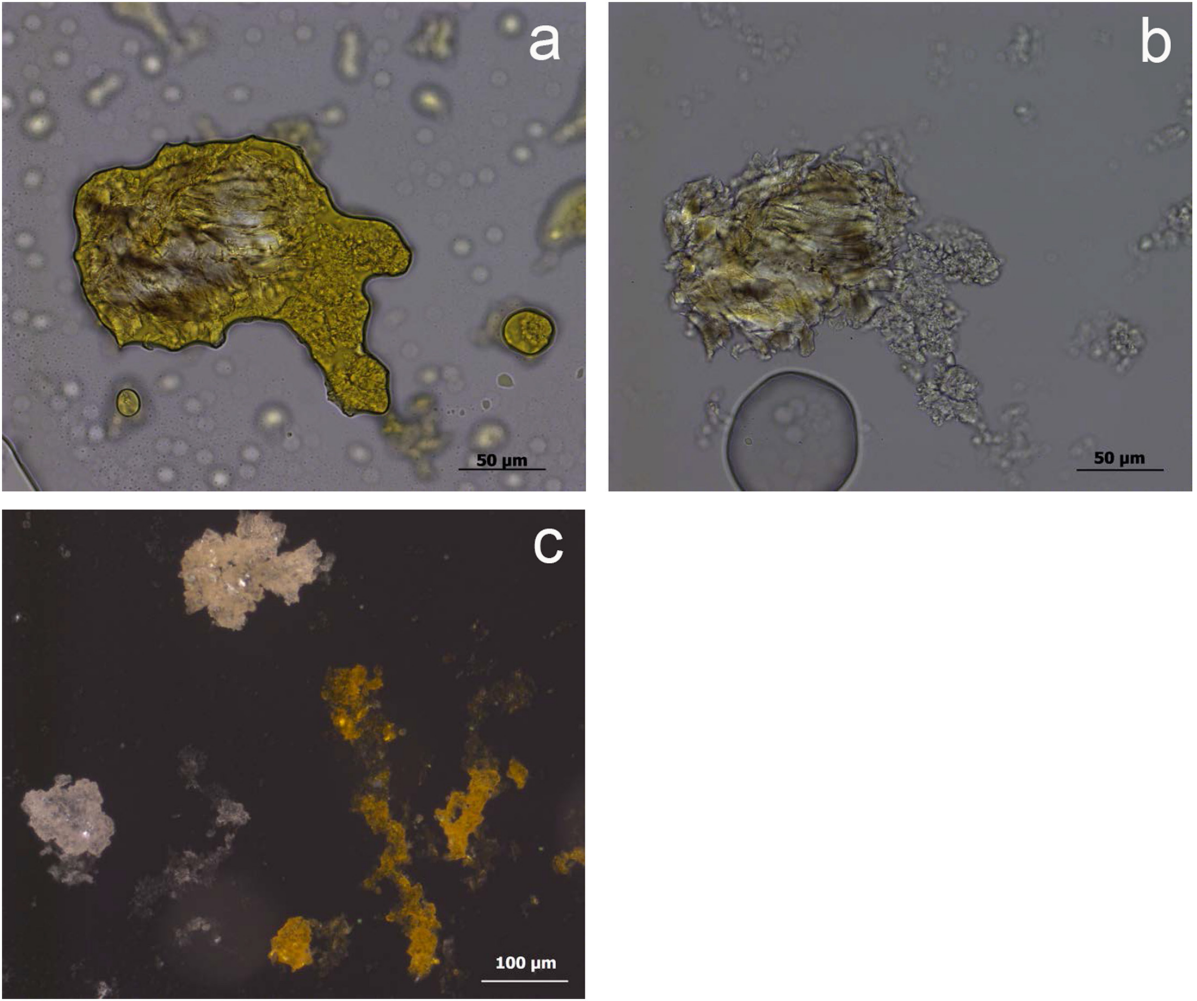
Re-evaluation of residues identified on BT16 (unused). **a)** Wood from retouching stained with orange G, after washing out not correctly (400x); **b)** Wood from retouching, after washing out correctly (400x); **c)** Wood partly stained with Orange G due to not washing out correctly the stain (200x).

Two freshly knapped and retouched artefacts (BT8 and BT16, both unused) were incorrectly interpreted as being used by all analysts. In each case, two different types of organic hammer were used for knapping and for retouch (antler and wood). Surprisingly, Analyst 1 and 2 interpreted these flakes as having been used for the processing of animal material. For BT8, this is not a surprise given other examples of antler retouch residues being mistaken for use residues and the other residue evidence that is present on this flake (see below). For BT16, it seems that wood residues were mistaken for animal material (Fig 4). Residues looked globular and amorphous with a yellow tinge under low magnification, making it difficult to identify wood or other plant tissue. Re-examination and comparison with other reference examples confirm that the morphological appearance of these residues is indeed similar to smeared wood tissue. However, the smearing obscures cell walls and makes identifications particularly difficult, stressing the importance of extracting the residue to make the internal structure apparent. Problematically, the residue extractions stained positive for collagen during the blind test. Re-examination showed that the positive result was due to incomplete rinsing of the stain (Fig 5). Only for one analyst did the distribution of antler retouch residues on a potential working edge and wood residue from production on the proximal extremity lead to the interpretation that this flake had been hafted and used (BT8 / Analyst 2, see below).

**Fig 6:**
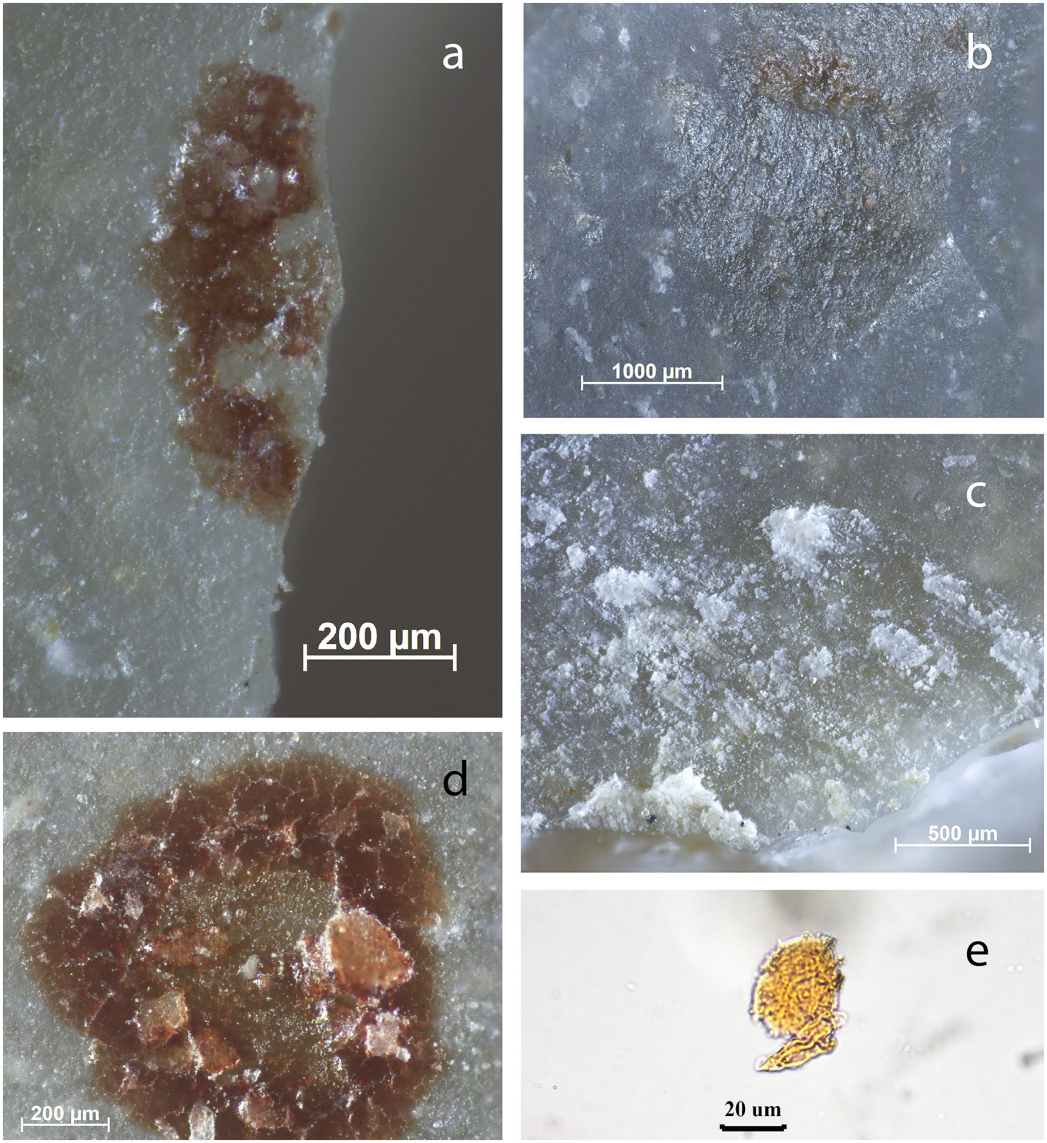
Assumed use-residues identified on BT8 (unused) as described during blind test. **a)** Blood residue on the distal retouched end of the flake; **b)** fatty/greasy film with blood; **c)** white amorphous residue (*cf*. bone) smeared perpendicular/ diagonal to edge; **d)** blood residue with “mud-cracked” appearance; **e)** collagen residue stained with Orange G, transmitted light.

While flake production and retouch may lead to residues resulting from the direct contact with the hammer, the knapper can also cause unexpected residues, as was the case for BT8 (Fig 6). This confusion was not anticipated, but apparently the knapper had cut himself during tool production leaving some blood on the stone tool. This small blood residue in combination with (1) a greasy, white amorphous residue (attributed to bone) smeared perpendicular to the edge, and (2) apparent collagen, which stained positive with Orange G, resulted in the interpretation—shared by all three analysts—that this flake was used for animal processing. Instead it was an endscraper, retouched with antler, but unused. Upon revision of their interpretations, analysts agreed that they had not adequately examined the residue distributions and characteristics. A correct identification would have been possible based on the following criteria: (1) the blood residues were not smeared as they should have in the case of use–they occurred only as droplets; however, this is partially contradicted by BT10 (see below); (2) the blood was very low in abundance and not evenly distributed along a potential working edge; (3) the bone and blood were not mixed in occurrence; the bone residue smears were separated from one another by several millimetres instead of being more or less continuous in distribution. Theoretically, the blood droplets could have been identified as human and more likely related to production, but in an archaeological setting, such an identification and conclusion would be far more difficult.

**Fig 7:**
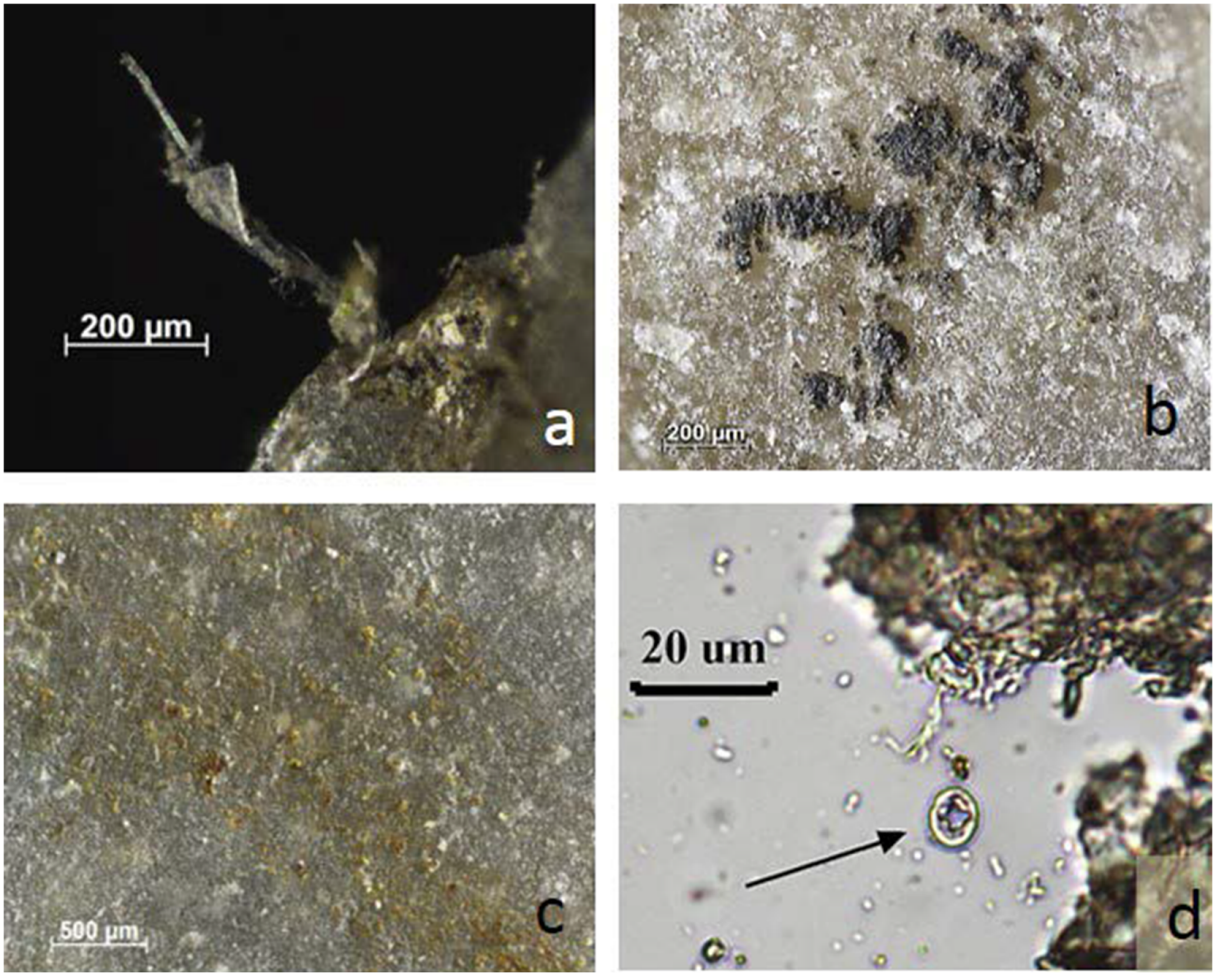
Residues on BT25 (unused) as described during blind test. **a)** Plant fibre on distal end of the ventral surface; **b)** unidentified, black tacky material (possible haft-residue) on ventral edge; **c)** sediment and organic fibres (*cf*. wood) occurring in an isolated region on the ventral surface, with evident directionality; **d)** starch grain and amorphous plant tissue removed from the same region as in c, along the edge on the ventral surface.

Incidental contact with various materials also resulted in wrong interpretations. Incidental contact may happen during production, as was the case for BT25, a freshly knapped blade that was dropped on organic-rich soil during knapping (Fig 7). Aside from the occurrence of dispersed plant fibres, soil contact also resulted in the deposition of other organic fibres with a directional pattern, at least one starch grain and some amorphous plant tissue. The organic fibres even tested positive with Orange G by Analyst 1, indicating the presence of collagen, and in spite of the fact that a low frequency of residues was mentioned by the analysts, the flake caused confusion. It was, however, only wrongly identified as used by one analyst. Re-examination of the slides suggests that a brown orange pigment in the sediment might have interfered with interpretation of the stain reaction. Much more likely, the identified collagen and starch were simply constituents of the organic-rich soil.

**Fig 8:**
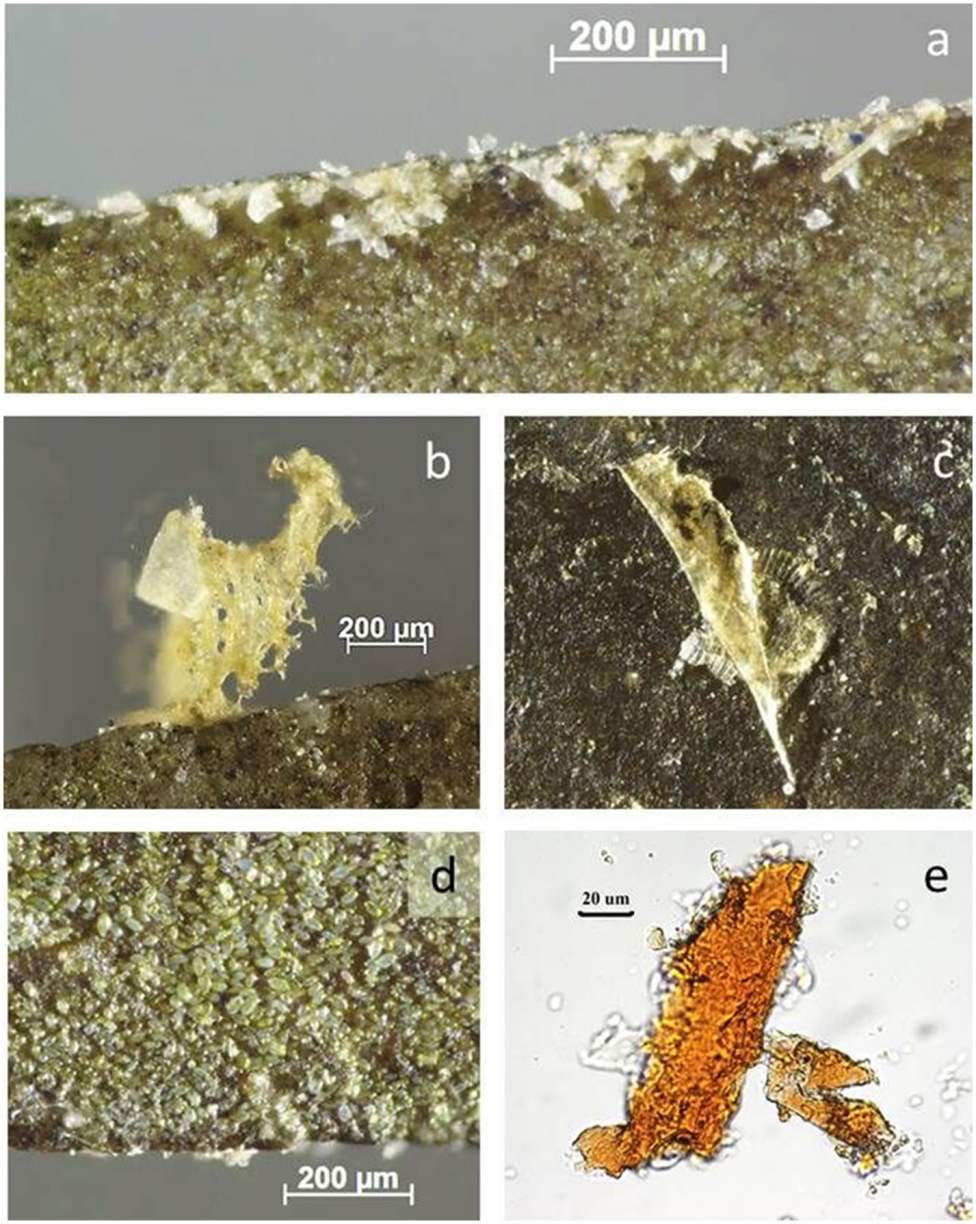
Residues on BT19 (unused, deposited in river) as described during blind test. **a)** Amorphous use-residue along Edge A, appearing as white translucent bundles of organic material; **b)** fatty animal collagen on unused edge; **c)** fish scale from the ventral proximal surface of the used edge; **d)** green algal spores, possibly contamination or part of the contents of the fish’s stomach; **e)** amorphous collagen tissue stained with Orange G.

The case above is an example of very low-pressure, incidental contact, but three more cases were tested involving various *depositional* contexts (Table 1) with a significant effect on the accuracy rates (Tables 5 and 6). The most remarkable case is BT19 that was deposited in a river bed (Fig 8). It was an unretouched flake, so retouch could not have caused confusion or false expectations regarding its use. Quite surprisingly, a fish scale proved to adhere to the stone tool surface as a result of a mere deposition of 2 months in a river (and in spite of it being rinsed). For all three analysts this immediately lead to the interpretation that the flake was used to process fish. In addition, white translucent bundles of organic material were observed along the edge as well as collagen. The green algal spores that were also macroscopically visible were interpreted as contamination or part of the contents of the fish’s stomach (Fig 8).

This example stresses the importance of considering the depositional context of all archaeological flakes examined for residues. Fish remains may preserve on a stone tool purely as the result of deposition in a fluviatile environment. It demonstrates that residues need to be sufficiently abundant and linked with other types of evidence before a stone tool can be reliably identified as having been used for fish processing. This result also implies that tools identified as having been used to process fish based on minimal residues may merit re-evaluation, given that incidental deposition of fish scales was unknown at the time [10].

**Fig 9:**
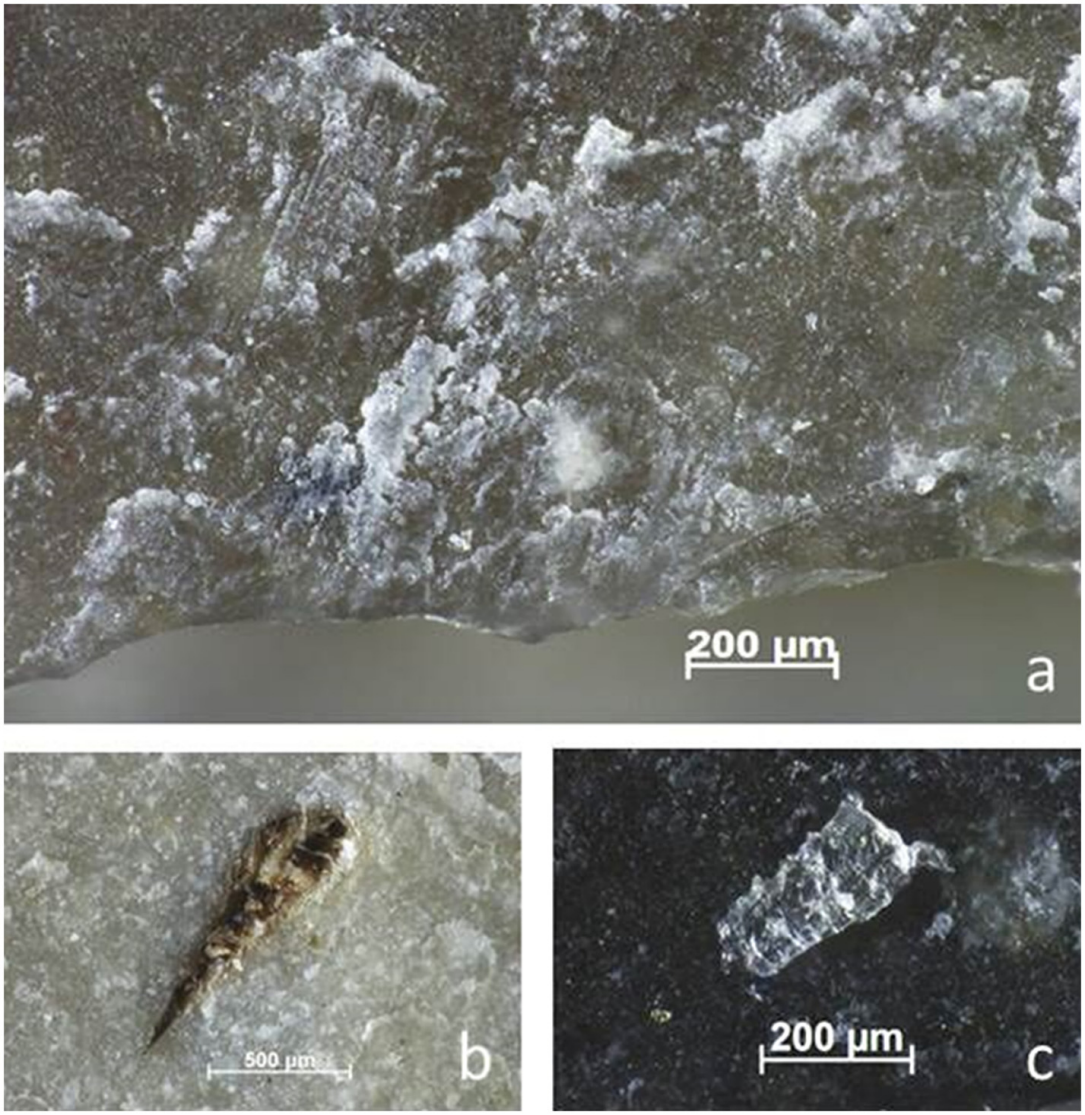
Residues identified on BT5 (unused, rolled in hide) as described during blind test. **a)** White use-residue with evident directionality (diagonal from edge) along the ventral used edge; **b)** unidentified solidified brown residue from the proximal surface, probably contamination; **c)** unidentified organic tissue along the ventral used edge.

Less invasive depositional scenarios may also contribute to misinterpretations as is exemplified by BT5, which was rolled in a piece of hide for 2 weeks (Fig 9). It was interpreted as used by all analysts, for animal processing by two analysts and for unidentified scraping by the third. However, it is not the depositional context, but the retouching that lies at the basis of the error in spite of the fact that the flake was retouched with sandstone and not with an organic hammer. The white smears resulting from this retouching were assumed to have an animal origin, due to which the two analysts decided to apply staining to examine the presence of collagen. Surprisingly, this staining with Orange G was negative with Analyst 1 and positive with Analyst 2. This suggests that a small residue from the hide may have been part of the latter extraction and not of the former. This minimal residue from the hide added to an already confusing pattern. In spite of different staining results, both analysts maintained their interpretation that the flake was used in scraping hard animal material based on the directional pattern in the white smearing. This demonstrates that even white residues left by sandstone hammers can mistakenly be attributed to an animal origin under incident light.

Burying an unused flake did not lead to as much misinterpretation as with the previous two cases. BT26 was interpreted as unused and possibly used by two analysts. A third analyst identified it as having been used to process organic material based on a low magnification analysis, but realised under high magnification that the occurrence of this organic material was very rare. Also the extractions did not pick up much organic material and the identification was thus reduced to uncertain. This is a good example of how an increasing level of detail in the analytical protocol has reduced the certainty with which an initial functional interpretation was made.

The final scenario of incidental residue deposition (N: 2) was whether residues could incidentally be deposited on a stone flake by just *lying nearby on the ground* when other tools are being hafted with resin (BT7) or with bindings (BT30). Both scenarios resulted in wrong interpretations by two analysts: woodworking by Analyst 2 for BT7, and scraping bone or skin by Analyst 3 for BT30. In both cases, it was not the depositional scenario that caused confusion, but the misinterpretations are surprising given that neither of the flakes were retouched. Analyst 2 observed wood tissue on BT7, but this residue did not indicate a used edge perhaps because it was of low abundance and/or minimally distributed. The problem here seems to be the over-interpretation of minimally distributed residues. BT30 caused confusion for all analysts and staining was applied by two of them. Apparently, production in this case had left a significant amount of wood residue on the platform and it is this residue that caused confusion. Such an error might be avoided when one realises that these residues could easily be the result of production. The wrong (though uncertain) interpretation of Analyst 3 is caused by the presence of crushed white residue on the distal extremity, while residue on the platform was correctly linked with production.

#### Impact of analytical protocol

When the predefined stepwise protocol is compared with the accuracy of the quick general screening, surprisingly few differences can be observed. Analyst 3 actually scores better in distinguishing used and unused flakes. However, few tool-use determinations were possible based on such a quick screening, and interpretations should be considered more as educated guesses with limited confidence. Nevertheless, it shows the relevance of including such a broad screening as a first analytical stage during which relevant samples are selected for a more detailed study. Including such a stage may in fact avoid errors based on incidental residues.

The first step in the predefined analytical protocol concerned an examination under incident light (up to 180x magnification). Both analysts who followed the procedure chose mainly to use the zoom binocular microscope with magnifications up to 180x, which proved very suitable and easy to use for this first stage of the residue analysis. Residue extraction, including the examination under transmitted light, did not prove to significantly affect the accuracy of distinguishing between used and unused flakes. Both approaches thus prove equally adequate for making these distinctions. Indeed, similar accuracies were obtained, but the use of more detailed techniques seemed to negatively affect the confidence level of the identifications: the increase in the detail and the ambiguous results of staining sometimes caused greater uncertainty. As a result, the number of wrong interpretations reduced as the number of non-identified flakes increased. This demonstrates that wrong identifications are in part the result of low residue abundance, incident residues or a confusing combination of residues, which upon closer examination resulted in the inability of analysts to truly identify whether the flake was used or not. However, understanding the problem suggests that it can be corrected in the future.

Staining did not contribute to correct identification of unused flakes at this stage of the analysis, on the contrary, it added to the confusion. Risk is high that incidental residues are stained and falsely linked with tool-use (Table 7). Consequently, a significant number of incorrect identifications were due to the fact that stained materials in reality had no link with used edges, but were from depositional contexts, production or hafting. The test results suggest that when one is not sure about the cause of a residue, a chemical technique will not necessarily aid in determining its origin. A stain may correctly assess the presence of collagen, cellulose or blood, like the stains used in this test, but this does not necessarily help in identifying what caused the transfer of residues and how they might be linked with a tool’s use or non-use. It may be that when only little parts or fragments of the material become stained, that the material is likely to be contamination and should not be considered to be linked with use. This was, for instance, the case with BT12 for which Analyst 2 maintained the uncertainty because the residue was not entirely stained.

**Table 7:** Summary of staining results per category of artefacts (*False positive*: something was stained that was not directly related to use, but to production, hafting or contamination; *correct negative*: no presence of the residue was correctly indicated). Analyst 3 did not apply staining.

STAINING—USED VS UNUSED	Nr of flakes	Analyst 1						Analyst 2					
		Nr of tools stained	Nr of stains applied	Stained use residue	Did not stain use residue	False positive	Correct negative	Nr of tools stained	Nr of stains applied	Stained use residue	Did not stain use residue	False positive	Correct negative
Used & handheld	**7**	3	3	2	1	0	0	2	2	1	0	1	0
Used & hafted	**4**	3	6	3	0	2	1	1	1	1	0	0	0
Used & buried	**3**	1	2	1	0	0	1	2	2	1	0	1	0
Deposited on material	**3**	3	5	0	1	3	1	3	3	0	0	2	1
Hafted & unused	**3**	3	4	0	0	4	0	2	2	0	0	0	2
Lying around when hafting	**2**	1	2	0	0	2	0	1	1	0	0	0	1
Freshly knapped & unretouched	**4**	2	3	0	0	3	0	2	2	0	0	2	0
Freshly knapped & retouched	**4**	3	4	0	0	4	0	3	3	0	0	1	2
**TOTAL**	**30**	**19**	**29**	**6**	**2**	**18**	**3**	**16**	**16**	**3**	**0**	**7**	**6**
***%***				***24***	***8***	***72***	***12***			***18*,*8***	***0***	***43*,*8***	***37*,*5***

#### Conclusion: distinction used / unused

It can be concluded based on this part of the test that both approaches commonly used in residue analysis are successful in identifying used flakes, but not so successful in identifying unused flakes. The main problems that were highlighted are the lack of recognition of production-related residues and the over-interpretation of small incidental residues. These problems can be corrected by understanding production residues and their potential directional nature and by a well-defined protocol in which the location, organisation and frequency of residues are critically examined. Attention needs to be devoted to the combination of residues on a tool’s edge and to their association within a functionally meaningful pattern (see also Lombard 2005, 2008). Residues need to be sufficiently abundant on an artefact’s edge before linking them with use (even if this factor is harder to control in archaeological conditions). It stresses the importance of performing a detailed in situ analysis of the residues before any extraction, with the above considerations in mind. Of course, an independent confirmation through use-wear analysis—preferable by a separate analyst—is likely to aid in correctly assessing the cause of residue deposition (e.g., Rots and Williamson 2004); and it may prevent errors and save time and costs, in particular when a broad use-wear screening is performed before the residue analysis.

Plant fibres are frequent incidental residues, while blood may come from the knapper. Deposition in fluviatile environments may cause a lot of contamination from the surrounding environment, including fish scales and algae. Retouch residue is generally confined to the outer edge and has a discontinuous distribution. It will evidently only occur in areas that are retouched, independent of the location of the working edge, and the residue generally occurs within the concavity of the negative left by a retouch flake, similar to what has been documented for retouch striations in wear studies [22,25].

While the analysis of sediment samples are frequently performed in the context of residue analysis to avoid an over-interpretation of residues that are derived from the depositional environment [1,41,67], such a procedure is not considered helpful to correct all problems identified in this part of the test. After all, most concerns were a consequence of residues that were deposited during production and retouch processes. Of course, we do agree with the necessity of including sediment samples in the analysis of archaeological tools [53,59,68] and the inclusion of the results in publications.

### Tool-use and used edges

For the examination of the accuracy rate of residue analysis in identifying tool use, only used tools are considered. There is no point in accumulating errors that were made on a preceding identification level. Tool residues were examined in situ (Table 8) and after extraction (Table 9). Three categories of used tools are considered and it is immediately clear that flakes that were buried after use caused most problems. Although these tools were used for relatively long durations (40 minutes– 1 hour) to allow an adequate build-up of residue, sediments acquired during the deposition phase of the tool’s use-life obscured any of the use-related residues, thus making them difficult to document (see also [69,70]. It was not entirely clear to the analysts whether the sediment was present as a result of use or burial. This problem would not arise with excavated archaeological assemblages as sediment would be present on all artefacts. However, it does imply that sediment may hinder the observation of residues on archaeological artefacts, necessitating at least some form of minimal and controlled cleaning (Cnuts and Rots in prep.).

**Table 8:** Summary of results based on in situ observation (up to 180x) for the identification of tool use per category of used artefacts (percentages calculated based on pieces correctly identified as having been used per analyst in order not to accumulate errors made on another identification level).

TOOL USE	Nr of used flakes	Analyst 1					Analyst 2					Analyst 3 / screening				
		*correct as used*	correct tool use	partially correct	wrong tool use	not identif	*correct as used*	correct tool use	partially correct	wrong tool use	not identif	*correct as used*	correct tool use	partially correct	wrong tool use	not identif
Used & hand-held	**7**	***6***	2	4	0	0	***4***	1	2	0	1	***7***	1	2	2	2
Used & hafted	**4**	***3***	2	1	0	0	***4***	2	1	1	0	***4***	2	0	0	2
Used & buried	**3**	***3***	1	0	2	0	***3***	0	2	1	0	***3***	0	1	0	2
**TOTAL**	**14**	***12***	**5**	**5**	**2**	**0**	***11***	**3**	**5**	**2**	**1**	***14***	**3**	**3**	**2**	**6**
***%***			***41*,*7***	***41*,*7***	***16*,*7***	***0*,*0***		***27*,*3***	***45*,*5***	***18*,*2***	***9*,*1***		***21*,*4***	***21*,*4***	***14*,*3***	***42*,*9***

Partially correct interpretations are those in which either the worked material or the use motion were incorrect.

**Table 9:** Summary of results based on transmitted-light observation for the identification of tool use per category of used artefacts (percentages calculated based on pieces correctly identified as having been used per analyst in order not to accumulate errors made on another identification level).

TOOL USE	Nr of used flakes	Analyst 1					Analyst 2				
		*correct as used*	correct tool use	partially correct	wrong tool use	not identif	*correct as used*	correct tool use	partially correct	wrong tool use	not identif
Used & hand-held	**7**	***6***	3	2	2	0	***4***	1	3	0	0
Used & hafted	**4**	***3***	2	1	0	1	***4***	2	1	1	0
Used & buried	**3**	***2***	1	0	2	0	***3***	0	2	1	0
**TOTAL**	**14**	***11***	**6**	**3**	**4**	**1**	***11***	**3**	**6**	**2**	**0**
***%***			***54*,*5***	***27*,*3***	***36*,*4***	***9*,*1***		***27*,*3***	***54*,*5***	***18*,*2***	***0*,*0***

Partially correct interpretations are those in which either the worked material or the use motion were incorrect.

Success rates vary significantly between about 20% up to 84% if partially correct interpretations are included. Correct and/or partially correct interpretations are slightly higher based on transmitted-light observations than based on in situ observations. Wrong interpretations remain under 20% for all three analysts based on in situ observations, which is an important result, but they are higher for Analyst 1 after analysis of extracted residues.

**Fig 10:**
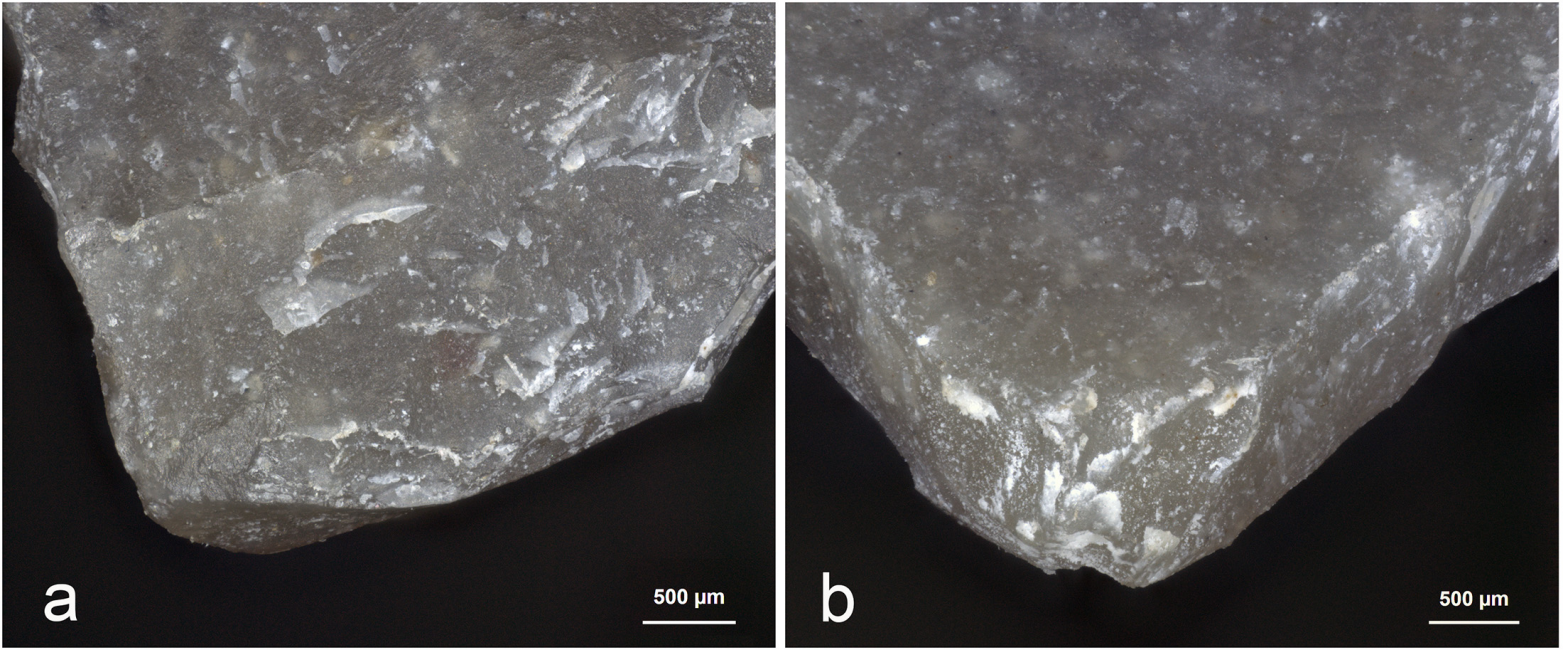
Re-evaluation of residue presence on BT15 (perforating antler) by comparison with experimental reference. **a)** Distal tip of BT15, used to groove antler for 25 minutes (50x); **b)** distal tip of exp. 69/06, used to groove antler for 30 minutes (50x). The tip of BT15 shows very few residue fragments in comparison to exp. 69/06, which is a consequence of the cleaning protocol.

**Fig 11:**
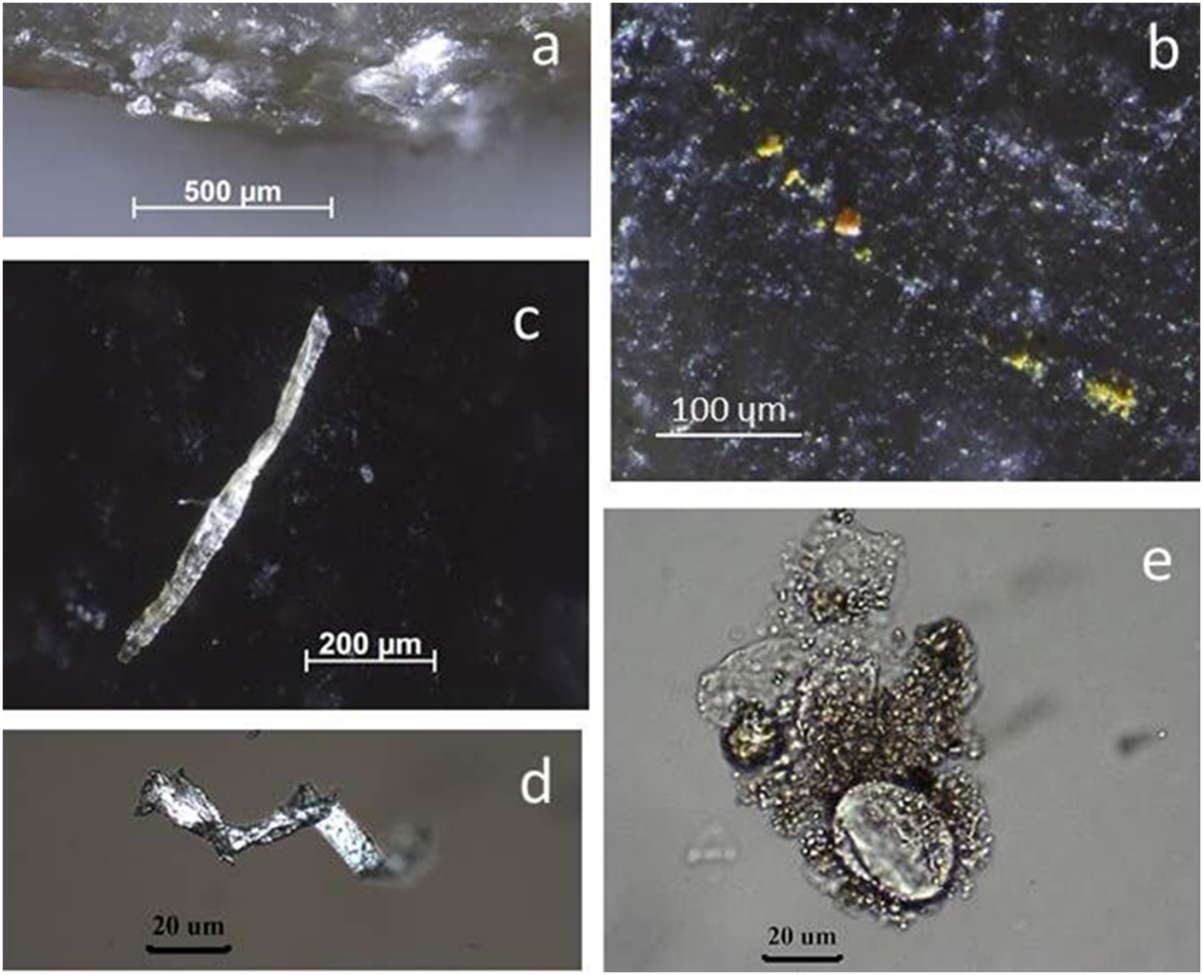
Residues identified on BT15 (perforating antler) as described during blind test. **a)** White, translucent, amorphous residue on artefact edge, possibly from use; **b)** yellow mineral residues, probably from incidental contact (non-use residue); **c-d)** plant tissue and cellulose fibre from tool surface, probably contamination; **e)** starch grain and cellulose fibres from extracted residue material sampled along the proximal right edge, transmitted light.

Only one used flake proved to be difficult for all analysts to recognise as having been used. Tool BT15 was used in the hand to work antler for 25 minutes. Probably the distal tip was not sufficiently examined and the area was not considered for taking the extractions. As such, the residue—which was not abundant (Fig 10)—was missed. Several other residues were observed on the flake, but none of them was considered to be sufficiently abundant to justify an identification of use (Fig 11).

#### Worked material

**Fig 12:**
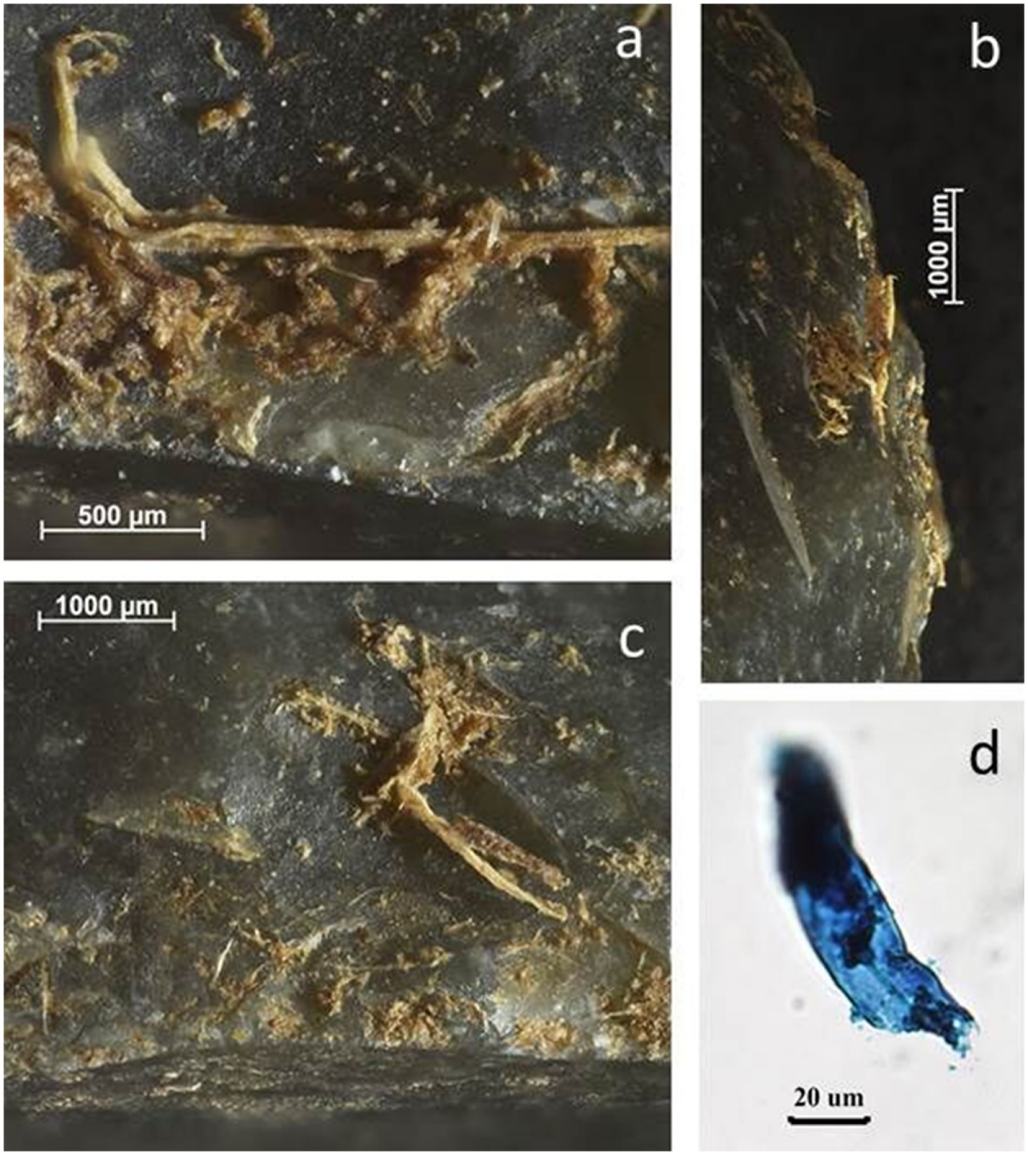
Use-residues identified on BT9 (scraping wood) as described during blind test. **a-c)** Woody fibres along tool edge, acquired during use; **d)** plant material from residue extraction, stained with Methylene Blue to confirm plant origin.

**Fig 13:**
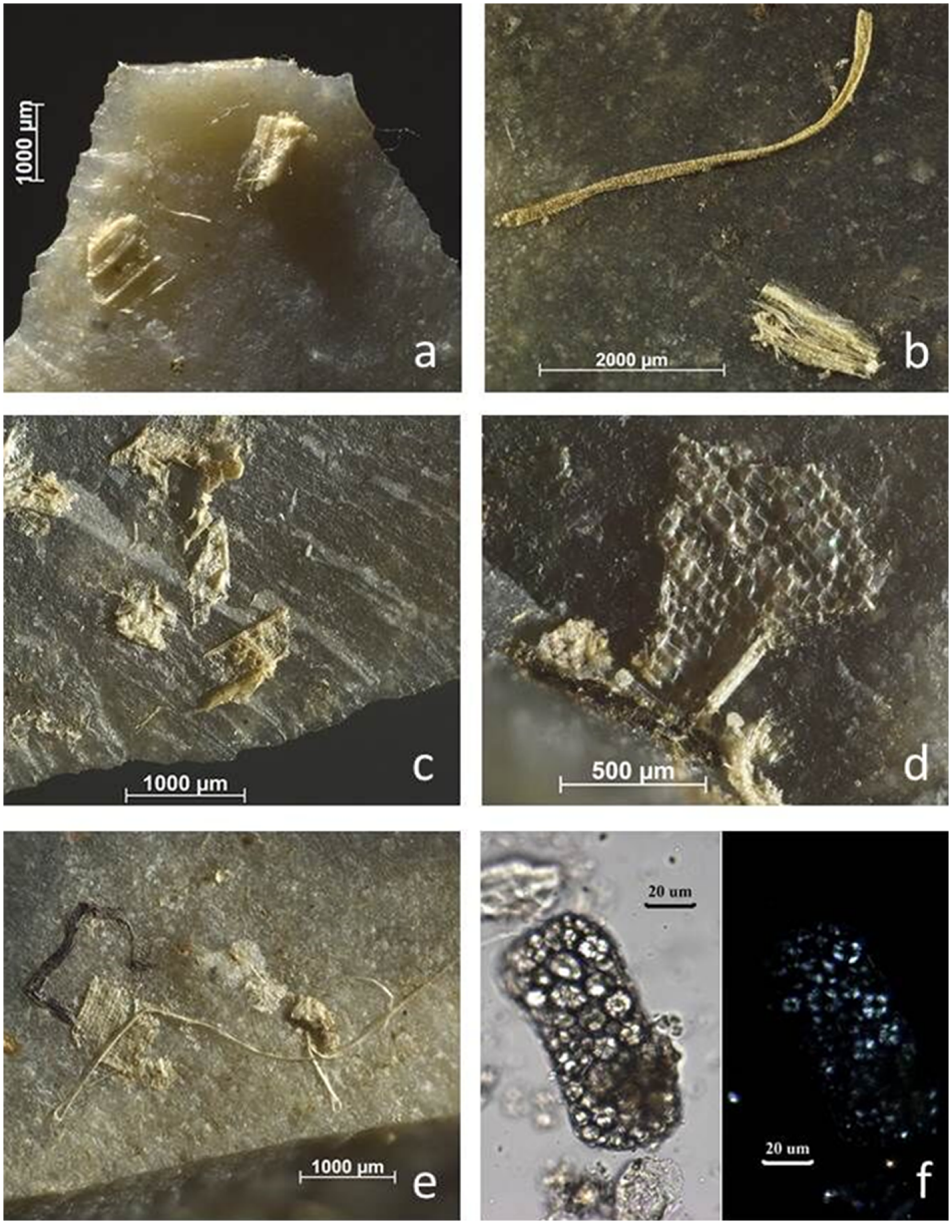
Use- and haft-residues on BT20 (processing tubers) as described during blind test. **a-c)** Macroscopically visible plant and wood fibres present at various locations across the artefact surface, including the used edge; **d)** plant residue with distinctive cell structure, present on the possibly hafted end of the tool; **e)** plant fibres and woody tissue on the non-used edge of the tool, artefact surface; **f)** bundles of starch grains removed from the working edge of the tool and photographed under transmitted light: left image in part-polarised light and right image in cross-polarised light to show distinctive extinction crosses.

Woodworking tools overall caused few problems (BT9, 11, 18, 28). The majority of them were identified correctly by all analysts, only BT11 (which had also been buried) and BT18 caused some partial problems. Residues were generally visually distinct with confirming staining results (Fig 12). Also the processing of tubers did not cause problems, mostly because large quantities of starch were recognised along the working edge of the tool (Fig 13).

The burying had a negative effect on the interpretation of BT24 and BT29 in spite of the fact that the most obvious sediment had been washed off under running water. As a result, Analyst 1 did not observe evidence of any other potential use. Based on the interpretations of Analyst 2 and 3, it is obvious that cutting bone leaves more marked evidence than cutting meat after a burial episode (which corresponds to the intensity and speed of their respective wear formation). Re-examination confirms that little residue was present on BT24 (e.g., spots of blood, collagen fibre), but that slightly more was visible on BT29. Future study is needed to assess whether this is due to degradation after burial or to the fact that sediment was trapped within the residue and may have been washed off during the cleaning protocol.

BT10 presents an interesting case of how different residues can cause a confusing picture. Two analysts had a problem with this tool. One problematic element was the presence of blood. Analyst 1 inferred meat cutting instead of hide cutting based on the combination of the blood residue with dried collagen material. However, the blood resulted from a knapping accident (as registered on experiment recording sheets), but in contrast to the case above (BT8), the blood on this flake was smeared due to subsequent handling and this smearing was used to argue for a use origin. Re-examination confirms that it indeed concerns human blood (based on the size of the platelets). The blood occurs on the edge opposite to the working edge, which explains why Analyst 1 inferred that both edges were used.

Similarly, butchering tool BT13 also caused problems with only one correct interpretation. The other analysts inferred hide cutting and plant cutting. The hide interpretation was based on the presence of hairs, which Analyst 2 considered to be typical for hide cutting, not butchering. Hairs can, however, also occur in the case of butchering.

Tools used on other worked materials had varying results. It was surprising that the cereal harvesting tool was not immediately recognised as such. Working hard animal materials proved to leave sufficiently diagnostic residues, on the condition that the used area was correctly identified: BT17 and 23 did not pose problems, while analysts did not see that the tip of BT15 was used (cf. supra).

#### Use motion

In residue analysis, use motions are generally inferred based on the directionality of residue smears. Scores are similar to the ones for the worked material identifications. In several cases, the tool-use interpretation was only partial (cf. Tables 8 and 9), but in more or less equal cases this concerned either an incorrect worked material or an incorrect use motion. Nevertheless, while worked materials are often provided but proved wrong, analysts were not always explicit about the exact use motion and restricted themselves to the more general category “processing”. This suggests that residue analysis may not be the ideal approach to examine use motions.

#### Working edge

No difference is made between the analytical methods for the identification of the used edge, as no difference in accuracy was observed (Table 10). Cases where two used edges were inferred instead of one (or the opposite) were considered to be partially correct. Analysts proved to have some difficulty in identifying the correct working edge. Results are very comparable between the analysts although this may not be reflected in the percentages of Table 10 as Analyst 2 and 3 had a large number of unidentified working edges. Overall, accuracy rates were higher in the case of hafted tools.

**Table 10:** Summary of results based on in situ observations (up to 180x) for the identification of used edge per category of used artefacts (percentages calculated based on pieces correctly identified as having been used per analyst in order not to accumulate errors made on another identification level). Partially correct means that when both edges were used only one was identified.

USED EDGE	Nr of flakes	Analyst 1					Analyst 2					Analyst 3 / screening				
		*correct as used*	correct used edge	partially correct	wrong used edge	not identif	*correct as used*	correct used edge	partially correct	wrong used edge	not identif	*correct as used*	correct used edge	partially correct	wrong used edge	not identif
Used & hand-held	**7**	***6***	1	3	2	0	***4***	0	0	1	3	***7***	2	1	1	3
Used & hafted	**4**	***3***	3	0	0	0	***4***	3	0	0	1	***4***	2	0	0	2
Used & buried	**3**	***3***	1	2	0	0	***3***	1	0	0	2	***3***	1	0	0	2
**TOTAL**	**14**	***12***	**5**	**5**	**2**	**0**	***11***	**4**	**0**	**1**	**6**	***14***	**5**	**1**	**1**	**7**
***%***			***41*,*7***	***41*,*7***	***16*,*7***	***0*,*0***		***36*,*4***	***0*,*0***	***9*,*1***	***54*,*5***		***35*,*7***	***7*,*1***	***7*,*1***	***50*,*0***

#### Analytical method

**Fig 14:**
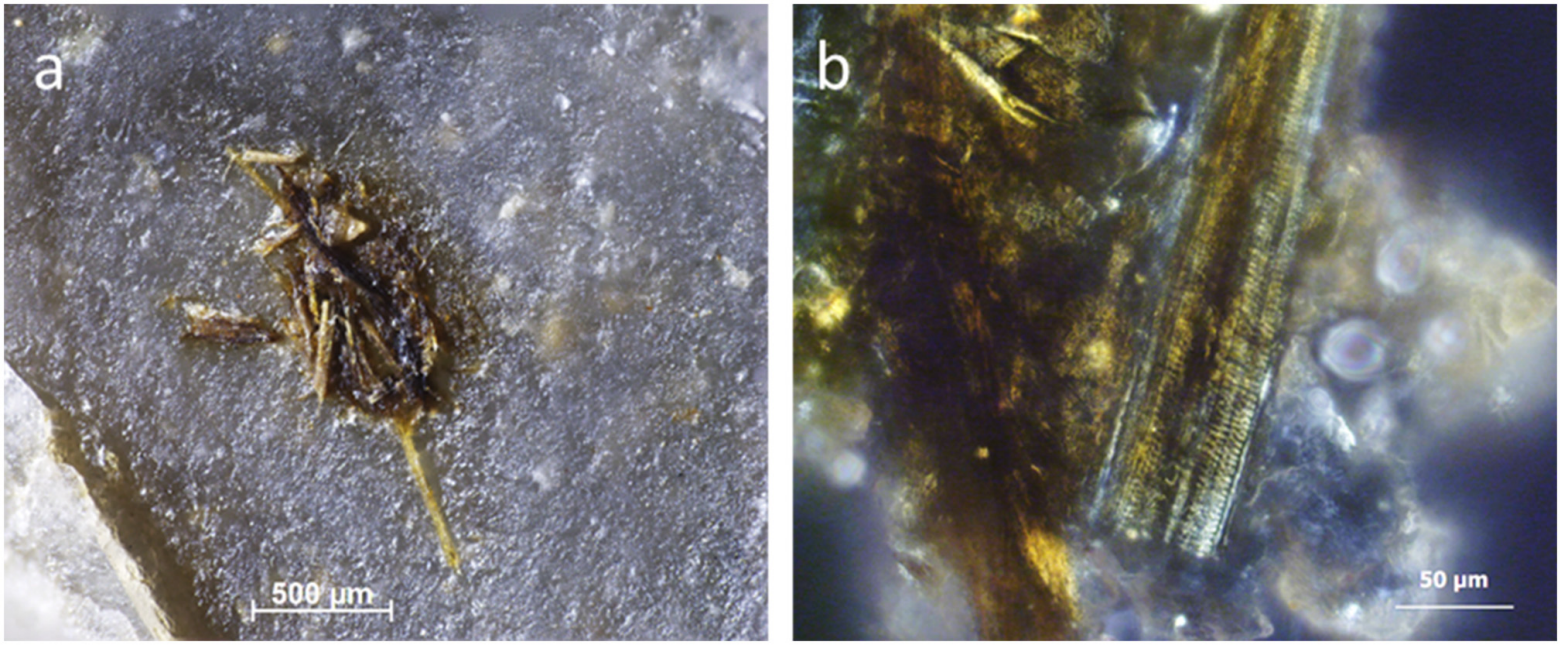
Residues identified on BT3 (processing fish) as described during blind test. **a)** Fish scale on the ventral surface of the flake, acquired during use; **b)** possible dried fish collagen, also on the ventral surface; acquired during use; **c)** fatty and greasy use residues (*cf*. blood and collagen), acquired during use; **d)** unidentified green fibre, possibly grass, with a fish scale beneath it.

The impact of the analytical method depends on what aspect of tool use is identified. While the impact is low for identifications of the use motion and working edge, it is more important for the worked material determinations. BT3 presents an example: the in situ analysis of Analyst 1 resulted in the identification “slicing grasses”, while extraction permitted the correct identification “slicing fish” (Fig 14). The tool was also correctly identified in the broad screening of Analyst 3. An increasing level of detail however had a negative influence on the identification of BT17, which was correct for both analysts when examined in situ, but incorrect for both when the extracted residues were evaluated. These results show that both approaches are equally suitable for identifying tool use even though all analysts agreed that they were more confident in identifying an individual residue under transmitted-light. Indeed, one needs to make a distinction between the confidence with which an individual residue is identified and the confidence with which tool use is inferred. While both approaches prove equally suitable for the latter, the increasing detail of residues that is visible under transmitted-light logically improves the former.

Staining confirmed some of the residue identifications and increased the certainty of the analyst interpretations. While staining did not help in determining residue origins or whether or not an artefact was used, the results suggest the relevance of staining for material identifications. Again, the above distinction between the identification of individual residues and the identification of tool use is relevant. Staining contributes to the former, but not to the latter. The test scores demonstrate that staining should only be applied at the end of a phased procedure, on tools for which other methods already suggested that they were used and what that use would be. Staining should be used for what it is intended: to assess the presence or absence of a certain compound (collagen, cellulose, blood) and not for confirming an unwarranted assumption.

### Prehensile mode

#### Hand-held vs hafted use

Most of the identifications of hand-held use were based on the absence of convincing evidence for hafting (Tables 11–13). Few positive identifications of hand-held use were made. This is because hafted use potentially leaves far more residues than is the case for hand-held use [25]. As Analyst 3 only performed a brief and quick screening, interpretations of the prehensile mode were only provided in the case of (supposedly) obvious indications (Table 11).

**Table 11:** Summary of results based on in situ observations (up to 180x) for the identification of prehensile mode per category of artefacts.

PREHENSILE MODE	Nr of flakes	Analyst 1				Analyst 2				Analyst 3 / screening			
		correct hafting	partially correct	wrong hafting	not identif	correct hafting	partially correct	wrong hafting	not identif	correct hafting	partially hafting	wrong hafting	not identif
Used & hand-held	**7**	1	0	6	0	1	0	5	1	2	0	1	4
Used & hafted	**4**	3	0	1	0	2	0	2	0	1	0	0	3
Used & buried	**3**	2	1	0	0	2	0	0	1	0	0	0	3
Hafted & unused	**3**	2	0	1	0	2	0	0	1	1	0	1	1
**TOTAL**	**17**	**8**	**1**	**8**	**0**	**7**	**0**	**7**	**3**	**4**	**0**	**2**	**11**
%		***47*,*1***	***5*,*9***	***47*,*1***	***0*,*0***	***41*,*2***	***0*,*0***	***41*,*2***	***17*,*6***	***23*,*5***	***0*,*0***	***11*,*8***	***64*,*7***

**Table 12:** Summary of results based on in situ observations (up to 180x) of the prehensile mode for the unused and non-hafted artefacts.

PREHENSILE MODE	Nr of flakes	*Analyst 1*				*Analyst 2*			
		*hand-held*	*hafted*	*none*	*not identif*	*hand-held*	*hafted*	*none*	*not identif*
Deposited on material	**3**	3	0	0	0	1	1	1	0
Lying around when hafting	**2**	0	0	1	1	1	1	0	0
Freshly knapped & unretouched	**4**	2	0	1	1	2	1	1	0
Freshly knapped & retouched	**4**	2	0	1	1	1	2	0	1
**TOTAL**	**13**	**7**	**0**	**3**	**3**	**5**	**5**	**2**	**1**
%		***53*,*8***	***0*,*0***	***23*,*1***	***23*,*1***	***38*,*5***	***38*,*5***	***15*,*4***	***7*,*7***

**Table 13:** Summary of results based on transmitted-light observations of extracted residues for the identification of the prehensile mode per category of artefacts.

PREHENSILE MODE	Nr of flakes	Analyst 1			Analyst 2		
		correct hafting	wrong hafting	not identif	correct hafting	wrong hafting	not identif
Used & hand-held	**7**	3	4	0	1	5	1
Used & hafted	**4**	3	1	0	1	3	0
Used & buried	**3**	2	0	1	2	0	1
Hafted & unused	**3**	2	1	0	1	1	1
**TOTAL**	**17**	**10**	**6**	**1**	**4**	**8**	**2**
%		***58*,*8***	***35*,*3***	***5*,*9***	***23*,*5***	***47*,*1***	***11*,*8***

The presence of potential hafting evidence created three types of confusion: either the hafting evidence was taken as an argument to suppose the tool was also used (e.g. BT22 –Analyst 1), or the hafting evidence was mistaken for use (e.g. BT14 –Analyst 3), or it was uncertain whether the residues were related to use or hafting (e.g. BT30). A greater dispersion of use-related residues also resulted in their wrong attribution to hafting, such as for the plant fibres and woody tissue on the hand-held tool BT20 (Fig 11).

Also residues from holding the tool in the hand during use caused mistakes: Analyst 1 identified the hand-held zone as the used zone on BT17. Similar to the formation of wear traces [20,25], residues derived from the worked material cover the hands during use and may result in residues being deposited in various locations. As observed in the case of wear, this is particularly the case for “dirty” activities, amongst which bone/antler working (cf. BT17).

Again, production-related residues were sometimes mistaken for hafting residues. An explicit example is BT8: for Analyst 2 the opposition between antler retouch residues on a potential working edge and wood residue from production on the proximal extremity lead to the assumption that this flake would have been used hafted. Another example is the blood residue of BT10 discussed above: it was located on the edge opposite the working edge and Analyst 2 interpreted it as resin due to their inexperience at the time with blood residue. Under low magnification, both may sometimes appear similar at first sight.

#### Analytical method

The analytical approach only affected the interpretation of the prehensile mode for 2 flakes (Analyst 2). Indeed, BT16 and BT28 were no longer considered as having been used hafted after analysis of the residue extractions, which is correct for BT16 even though the tool was mistakenly considered as having been used, but incorrect for the hafted tool BT28. It implies once again that both approaches are equally suitable for identifying the prehensile mode of stone tools.

**Fig 15:**
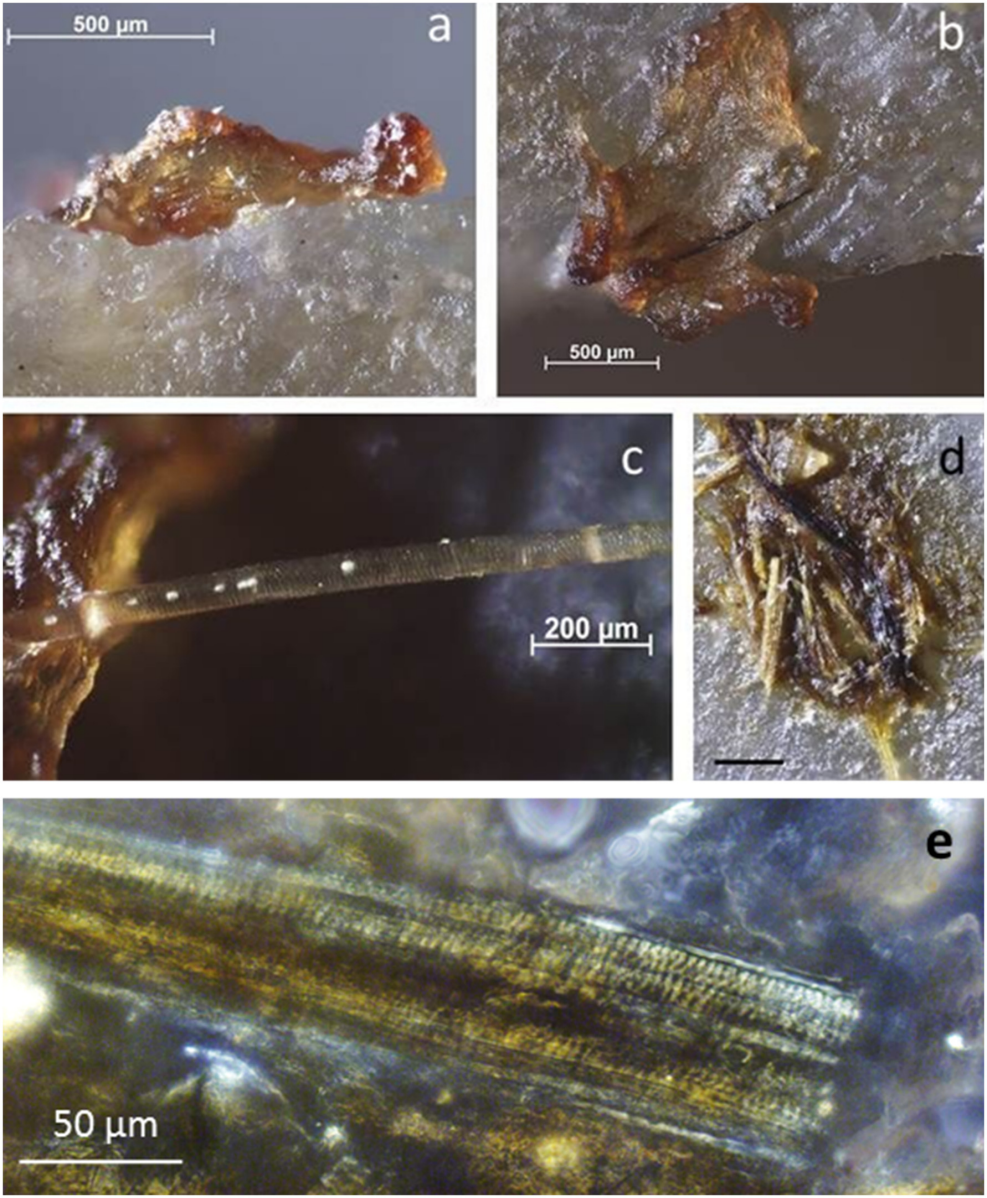
Use- and haft-residues identified on Blind Test Tool 13 as described during blind test (a-d) and during re-examination (e). **a-b)** Striated muscle tissue, blood and collagen residues on tool edges; **c)** hair fibre (*cf*. horse or deer) imbedded in muscle tissue on used edge, ventral surface; **d)** woody fibres possibly from hafting, dorsal surface; **e)** collagen fibre (not wood) after extraction during re-examination.

Extracting the residues for closer examination is however again proven important when it concerns a more accurate identification of an individual residue, as confirmed by analysis of BT13. On the stone tool, some visible residue was interpreted as being wood and it was attributed to hafting. However, this residue was not extracted during the test but only after the test had been completed. The re-examination confirmed that the fibres were in fact obvious collagen fibres and not wood (Fig 15). This indicates the importance of residue extractions for more accurate and reliable identifications.

## Discussion

While the results of this blind test indicate a difficulty of residue analysis in identifying unused artefacts and a particular used edge, use-wear blind tests have systematically shown the reliability of the method in identifying the used edge and whether an artefact was used or not. Therefore, the combination of both methods in a sequential procedure seems to guarantee a higher accuracy on different levels (cf. integrated or independent approaches). The presented blind test concerned residues only but combined with previous experiences of wear analysis (e.g., [71]) and results of previous integrated approaches (e.g., [11,48,54]), we believe that an ideal sequential procedure may be proposed (several elements of this procedure have been previously published by us or other analysts, e.g. [72] and see section 2 for other references). The degree to which such an ideal procedure can be put into practice depends on the individual assemblage or site.

As a first step, assemblages are best screened under low magnification for wear traces. This examination does not necessitate cleaning and residues are thus preserved. To avoid contamination, this analysis should be performed while wearing powder-free gloves. The low magnification analysis allows a quite reliable distinction between used and unused tools. In a second stage, residue analysis can be focussed on the likely used artefacts, preferably the ones with the highest potential and best preservation, by mapping all residues on the artefacts (cf. in situ analysis), evaluating their frequency and association, and critically assessing the cause of residue presence. Attention should be devoted to their association with fractures or other edge damage. In a third stage, relevant residues can be extracted with pipettes to allow more detailed identification, as with staining to obtain chemical confirmation. In a fourth stage, the artefacts can be submitted to a wear analysis that is likely to include a larger tool sample than the one considered for residues given the time-intensive nature of the latter. This stage may require that tools are cleaned and that residues will be removed. To prevent loss of residues, this stage may involve a full residue extraction of part or all surfaces in an ultrasonic tank with retention of the extracted residues in vials for further or future study and examination. This cleaning protocol may also be applicable when tools are not submitted to a wear analysis but need to be available for technological analysis.

While this sequential procedure may seem to be long, it actually guarantees a more time-efficient process by adapting the sample size to each analytical phase. After all, both wear and residue analyses are time-intensive methodologies. It also compensates for the small average sample sizes of most residue studies [73]. We therefore believe that this procedure in which residue and wear analysis are combined (in an integrated or independent way) may be the best guarantee for reliable functional identifications on reasonably large samples.

## Conclusion

Blind testing is a useful way to progress methodologically by highlighting interpretative problems and potential. Previous residue blind tests have focussed on the correct identification of residues and have contributed to improvements in the accuracy of residue interpretations to identify tool use. A first objective of the blind test discussed here was to examine whether analysts were able to make sense of residues resulting from different causes, realistic within the framework of the lifecycle of a stone tool (or unused artefact) and its subsequent deposition. A key issue was whether these processes could result in the deposition of residues that could be mistaken for use residues. This indeed proved to be the case and incorrect interpretations proved to be more common than anticipated when first designing the test. In nearly all cases misinterpretations of tool function resulted from either ignorance about the scale of extensive residue deposition from tool production and retouch; or from over-interpretation of low residue abundance. In our opinion, this increasing awareness of these issues is a crucial step in improving accuracy rates in determining use-related residues. Errors can be avoided by adequately mapping the location of residues (cf. in situ approach advocated by several analysts), by critically examining residue association and frequency, and by considering all possible causes of residue deposition (during the life cycle of a tool and after discard).

A second objective of the test was to evaluate the accuracy in identifying tool-use of the two approaches in residue analysis that are most commonly applied: an in situ analysis and an analysis of extracted residues. The test results indicate no relevant difference between approaches for accurately distinguishing used and unused tools, or for identifying tool use and prehensile mode. A difference was only observed in the accuracy and confidence with which an individual residue was identified, as extraction allowed the observation of more distinctive traits. Staining only contributed to the accuracy of individual residue identifications and proved to play no role in distinguishing between used and unused tools or in identifying tool use.

In situ analysis and residue extraction are not mutually exclusive approaches; on the contrary, they can, preferably, be combined. Given the importance of residue patterning, it is for instance clear that ultrasonic bath extractions without initial in situ screening should be avoided at all costs. A residue analysis preferably first involves a thorough mapping and examination of the in situ residues, followed by localised pipette extractions of particular residues for more detailed observation under transmitted-light. This procedure seems to provide the best chances for a reliable identification of tool use (in a broad sense) and individual residues.

Up to now, attention in residue analysis has been focussed a lot on correctly identifying residues, which is an essential step. Different new methods have been proposed, including chemical protocols like staining, SEM-EDX, GC-MS and FTIR to aid in a correct identification. Now it is time to also find ways to discriminate between different causes or processes of residue transfer. It is a methodological evolution that use-wear analysis had to go through. While initially, all traces were attributed to use from the perspective that wear traces do not form so easily on stone tools, later studies demonstrated that confusion may exist with other trace causes such as production [21,22], prehension [23,74] or hafting [25,71]. Similar to wear traces, production residues can be distinguished based on their organised pattern in relation to a technological feature (platform, retouched edge). Such residues can show smearing and a directional pattern, which is not to be confused with use, but they generally remain confined to the outer edge of the artefact. Hafting residues can be distinguished based on their location opposite a used edge. In all cases, an individual or isolated residue should not lead to the conclusion that a tool was used. A relatively high residue frequency and concentration in a particular part of the edge is usually required for a reliable interpretation. Preferably, different types of residue, all potentially derived from one source, occur in the same area (e.g. animal fibres combined with fat and collagen). The extension of the residues can be very large and may cover large parts of the tool or of the tool portion that sticks out of the haft.

While more quantitative analytical methods, like SEM-EDX, GC-MS, Raman or FTIR, are important to aid in correct residue identifications, their contribution to distinguishing between residue causes is limited in their capacity to determine tool function (although quantitative mapping of residue distributions holds promise). After all, it is the archaeologist as residue analyst who has to argue for a link between an observed residue and its functional significance, based on multiple lines of evidence including site context, technology, wear traces and taphonomic factors. As an initial stage, few alternatives to optical microscopy exist to try and obtain a reliable interpretation of residue origins. Study of residue frequency and abundance are valid ways to identify likely evidence of use, but as previously argued by other analysts, integration with the examination of wear traces (often hidden beneath the residues) seems essential—at least in the current stage of methodological development.
