# Correction: Making Sense of Residues on Flaked Stone Artefacts: Learning from Blind Tests

**DOI:** 10.1371/journal.pone.0178311

**Published:** 2017-05-18

**Authors:** Veerle Rots, Elspeth Hayes, Dries Cnuts, Christian Lepers, Richard Fullagar

The image for Fig 14 is incorrect. Please see the correct Fig 14 here.

**Fig 14:**
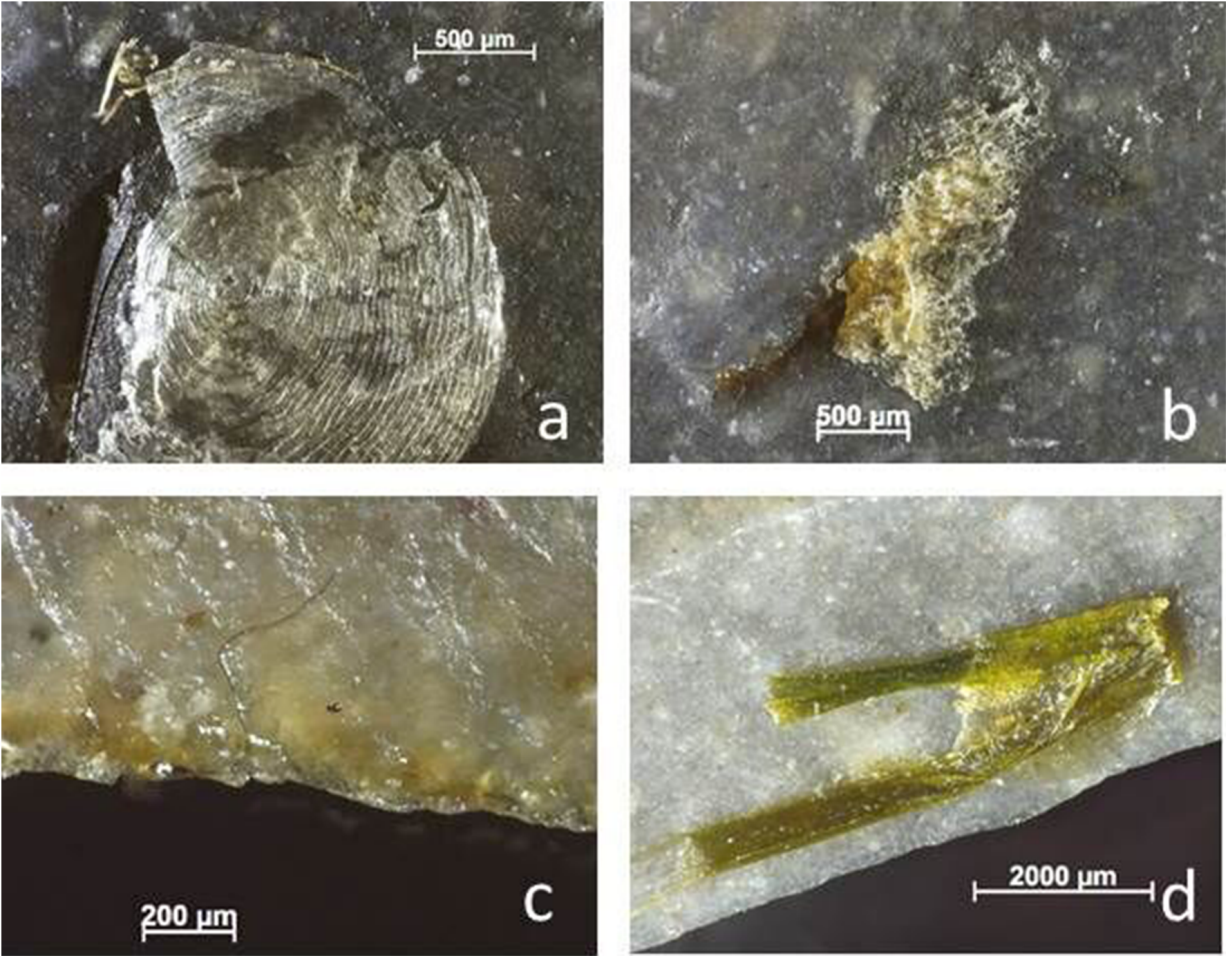
Residues identified on BT3 (processing fish) as described during blind test. a) Fish scale on the ventral surface of the flake, acquired during use; b) possible dried fish collagen, also on the ventral surface; acquired during use; c) fatty and greasy use residues (*cf*. blood and collagen), acquired during use; d) unidentified green fibre, possibly grass, with a fish scale beneath it.
